# *Brucella* MucR acts as an H-NS-like protein to silence virulence genes and structure the nucleoid

**DOI:** 10.1128/mbio.02201-23

**Published:** 2023-10-17

**Authors:** Ian S. Barton, Zhongqing Ren, Connor B. Cribb, Joshua E. Pitzer, Ilaria Baglivo, Daniel W. Martin, Xindan Wang, R. Martin Roop

**Affiliations:** 1 Department of Microbiology and Immunology, Brody School of Medicine, East Carolina University, Greenville, North Carolina, USA; 2 Department of Biology, Indiana University, Bloomington, Indiana, USA; 3 Department of Environmental, Biological and Pharmaceutical Sciences and Technologies, University of Campania “Luigi Vanvitelli”, Caserta, Italy; University of Washington, Seattle, Washington, USA

**Keywords:** MucR, *Brucella*, virulence, H-NS, H-NS-like, counter-silencer, nucleoid structuring, Hi-C, ChIP-seq

## Abstract

Histone-like nucleoid structuring (H-NS) and H-NS-like proteins serve as global gene silencers and work with antagonistic transcriptional activators (counter-silencers) to properly coordinate the expression of virulence genes in pathogenic bacteria. In *Brucella*, MucR has been proposed as a novel H-NS-like gene silencer, but direct experimental evidence is lacking. Here, we show that MucR serves as an H-NS-like silencer of the *Brucella abortus* genes encoding the polar autotransporter adhesins BtaE and BmaC, the c-di-GMP-specific phosphodiesterase BpdB, and the quorum-sensing regulator BabR. We also demonstrate that the MarR-type transcriptional activator MdrA can displace MucR from the *btaE* promoter, supporting the existence of MucR counter-silencers in *Brucella*. Moreover, our chromatin immunoprecipitation (ChIP)-seq analysis identified 546 MucR enrichment peaks along the genome, including in the promoters of the genes encoding the Type IV secretion machinery and effectors and the quorum-sensing regulator VjbR. Importantly, MucR ChIP-seq peaks overlap with the previously described binding sites for the transcriptional activators VjbR, BvrR, and CtrA suggesting that these regulators serve as MucR counter-silencers and work in concert with MucR to coordinate virulence gene expression in *Brucella*. In addition, using chromosome conformation capture (Hi-C), we show that like H-NS in *Escherichia coli*, MucR alters the global structure of the *Brucella* nucleoid. Finally, a copy of the *E. coli hns* rescues the distinctive growth defect and elevated *btaE* expression of a *B. abortus mucR* mutant. Together, these findings solidify the role of MucR as a novel type of H-NS-like protein and suggest that MucR’s gene-silencing properties play a key role in virulence in *Brucella*.

**IMPORTANCE:**

Histone-like nucleoid structuring (H-NS) and H-NS-like proteins coordinate host-associated behaviors in many pathogenic bacteria, often through forming silencer/counter-silencer pairs with signal-responsive transcriptional activators to tightly control gene expression. *Brucella* and related bacteria do not encode H-NS or homologs of known H-NS-like proteins, and it is unclear if they have other proteins that perform analogous functions during pathogenesis. In this work, we provide compelling evidence for the role of MucR as a novel H-NS-like protein in *Brucella*. We show that MucR possesses many of the known functions attributed to H-NS and H-NS-like proteins, including the formation of silencer/counter-silencer pairs to control virulence gene expression and global structuring of the nucleoid. These results uncover a new role for MucR as a nucleoid structuring protein and support the importance of temporal control of gene expression in *Brucella* and related bacteria.

## INTRODUCTION

*Brucella* spp. are important pathogens of humans and animals, causing abortion and infertility in their natural hosts and brucellosis in humans, which is one of the world’s most widespread zoonoses (1). The brucellae are predominately intracellular pathogens and must properly respond to this environment and subvert host immunity to establish a replicative niche inside macrophages and placental trophoblasts (2, 3). It is well appreciated that stimulus-responsive transcriptional regulators are essential in *Brucella* spp. for the coordination of host-associated gene expression and adaptation to their intracellular niche (4–6).

Proper coordination of host-associated behaviors is critical for bacteria that are pathogens or symbionts. Signal transduction pathways allow environmental signal integration into complex transcriptional networks and result in rapid niche-specific gene regulation through the activity of transcriptional regulators (7, 8). Recent evidence suggests that transcriptional repressors known as ‘gene silencers’ add an important layer to this regulation by preventing the gratuitous expression of virulence or symbiosis genes until this repression is overcome by antagonistic transcriptional activators known as ‘counter-silencers’ in response to host-specific environmental cues (9–12).

The histone-like nucleoid structuring (H-NS) protein plays an important role in coordinating the transcriptional expression of host-associated functions in many bacteria (12–15). H-NS acts as a global transcriptional ‘gene silencer’ by binding to AT-rich regions within promoters and preventing transcriptional activation through DNA stiffening (16) and/or bridging activities (17). H-NS-mediated transcriptional silencing is overcome through competition with ‘counter-silencers’ that bind to DNA regions overlapping or near H-NS binding sites and dislodge H-NS through mechanisms that are not well understood (15, 18–21). H-NS-like proteins such as MvaT in *Pseudomonas* (22), Lsr2 in *Mycobacterium* (23), and Rok in *Bacillus* (24) share minimal sequence or structural homology with H-NS but also act as global transcriptional repressors through binding and oligomerizing on adenine/thymidine (AT)-rich DNA sequences in bacteria that do not possess H-NS orthologs. Like H-NS, these H-NS-like proteins have been shown to be important for the survival and proliferation of these bacteria within their respective hosts (25) and functional counter-silencers have been identified for the H-NS-like proteins (26). In addition to coordinating the proper expression of host-associated genes, H-NS and the H-NS-like proteins also play important roles in organizing and structuring bacterial nucleoids (27) and preventing the toxic expression of genes acquired by horizontal gene transfer (HGT) (28, 29).

MucR is a prokaryotic zinc finger protein that serves as a global regulator in the α-proteobacteria where it acts predominantly as a transcriptional repressor (30). MucR homologs control many genes required for the symbiotic and pathogenic interactions of these bacteria with their respective plant and animal hosts (8, 31–34), and two paralogs MucR1 and MucR2 play important roles in coordinating progression through the cell cycle in *Caulobacter crescentus* (35). Studies of multiple MucR-regulated genes and global chromatin immunoprecipitation (ChIP-seq) analysis indicate that MucR binds to AT-rich regions with little consensus in the promoters of the genes it regulates (35–38), and mutational studies have shown that oligomerization is required for MucR’s regulatory activity (39, 40). These properties in combination with the fact that characterized MucR binding sites contain multiple TA ‘steps’ (36, 38) and the observation that MucR binds to the minor groove of DNA (36) have led to the proposition that MucR represents a novel type of H-NS-like gene silencer (41, 42) that plays an important role in orchestrating the proper temporal expression of genes in the α-proteobacteria (43) and protecting them from the potentially toxic expression of genes acquired by HGT (42).

MucR is required for the wild-type virulence of *Brucella abortus*, *Brucella melitensis*, *Brucella canis,* and *Brucella ovis* in mice, and transcriptomic and proteomic studies have uncovered regulatory links between MucR and genes encoding multiple virulence determinants including those involved in Type IV secretion, quorum sensing, cyclic diguanosine monophosphate (c-di-GMP) signaling, adhesion, lipopolysaccharide (LPS), cyclic-β-glucan, flagella and outer membrane protein (OMP) biosynthesis, and iron acquisition (34, 44–47), but the precise nature of these regulatory links and how they contribute to virulence are largely unresolved. Here, we combine ChIP-seq, genetic, and biochemical approaches to provide support for the proposition that the *Brucella* MucR functions as a bona fide H-NS-like gene silencer that works in concert with antagonistic counter-silencers to ensure the proper temporal regulation of genes encoding important virulence determinants. Using chromosome conformation capture (Hi-C), we also demonstrate that MucR plays an important role in maintaining nucleoid structure in *B. abortus* 2308. Finally, we show that the *Escherichia coli hns* gene can rescue elevated virulence gene expression and the characteristic growth defect displayed by an isogenic *B. abortus mucR* mutant. Altogether, these findings strongly support the proposition that MucR is a novel type of H-NS-like gene silencer and that its function as an H-NS-like gene silencer plays a major role in its contribution to virulence.

## RESULTS

### MucR is a global H-NS-like gene silencer in *Brucella*

**Fig 1:**
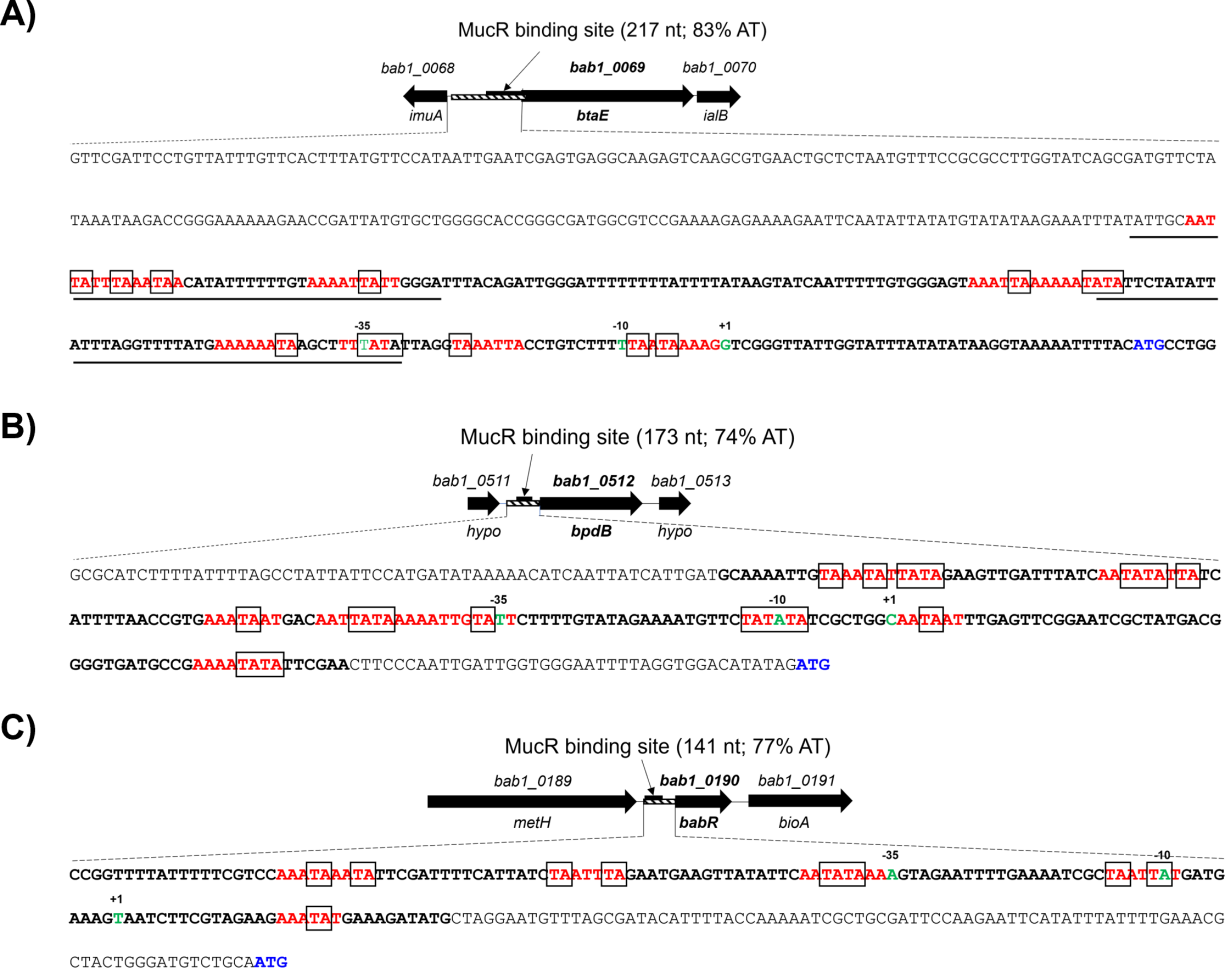
MucR binding sites in the *B. abortus* 2308 *btaE* (**A**), *bpdB* (**B**), and *babR* (**C**) promoters identified by deletion mapping (Fig. S1). The cross-hatched bar on the schematic shows the DNA region evaluated in EMSAs. Nucleotides shown in bold are the MucR binding regions and those shown in red are AT-rich regions with multiple TA “steps” (shown in boxes). The –35, –10, and +1 start sites for *btaE*, *bpdB*, and *babR* are shown in green, and the start codons are shown in blue. The transcriptional start sites for *btaE* (49) and *babR* (50) were determined by primer extension, and the transcriptional start site for *bpdB* was determined by differential RNA-seq (Caswell and Roop, unpublished data). Binding sites identified for MdrA in the *btaE* promoter region are underlined (49).

Previous microarray analysis indicates that MucR regulates >60 genes in *B. abortus* 2308 (34) with most genes being upregulated in a *mucR* mutant suggesting that MucR acts primarily as a global transcriptional repressor. MucR’s capacity to repress the genes encoding the autotransporter adhesins BtaE, BtaF, and BmaC, the quorum-sensing regulator BabR (aka BlxR), the c-di-GMP-specific phosphodiesterase BpdB, a putative transposase protein (TnsA), a hypothetical protein (BAB1_1035), and the *mucR* gene itself has been independently verified by qPCR or reporter assays and direct interactions of MucR with the promoter regions of these genes have been demonstrated here and previously in electromobility shift assays (EMSAs) (34, 37, 39) (Fig. S1). Analysis of the MucR binding sites localized in the promoters of the *btaE* (Fig. 1A), *bpdB* (Fig. 1B)*,* and *babR* (Fig. 1C) genes by deletion mapping (Fig. S1) identified binding regions that are very AT-rich (74%–83% AT) in comparison to surrounding regions and the overall 42% AT composition of the *B. abortus* 2308 genome. In addition, we found that the MucR binding sites detected in these regions are extended in length, ranging from 141 to 217 nt, and contain multiple TA ‘steps’ (48). Similar AT-rich regions are also present upstream of the *btaF*, *bmaC*, *tnsA*, *bab1_1035,* and *mucR* genes although the precise locations of the MucR binding sites relative to the transcriptional start sites for these genes have not been experimentally determined. These characteristics coupled with the fact that MucR oligomerization is required for its ability to repress *btaE, bpdB,* and *babR* in *B. abortus* 2308 (39) are consistent with MucR’s proposed role as a novel type of H-NS-like gene silencer (41, 42).

**Fig 2:**
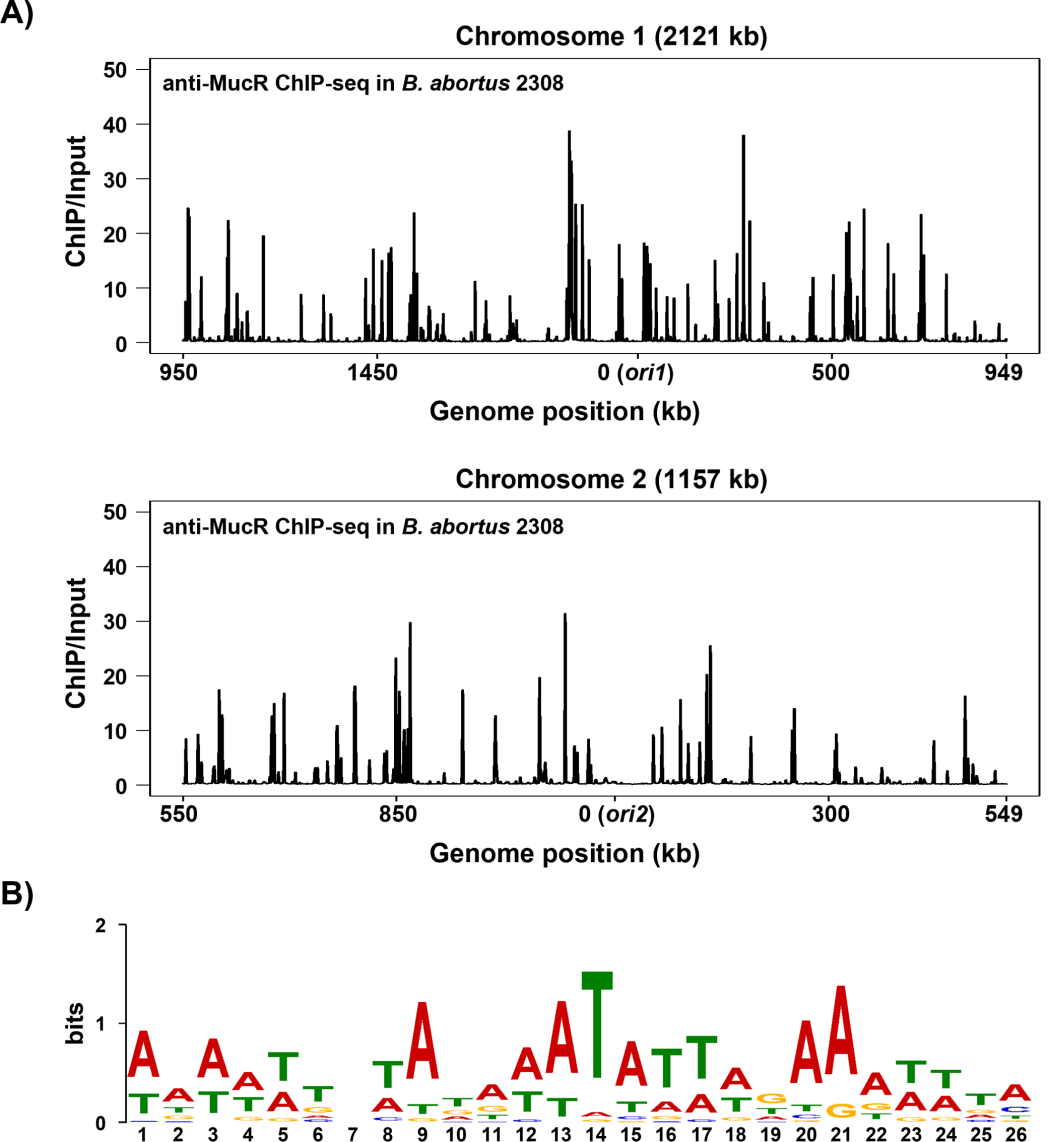
(**A**) Distribution of MucR binding sites detected by ChIP-seq analysis on Chromosomes 1 and 2 of *B. abortus* 2308. The x-axis shows genome position in kilobases (kb). The y-axis represents the fold enrichment (ChIP/Input). For each sample, the sequencing reads at each position were normalized to the total number of reads before plotting. The data were plotted in 1 kb bins. Origin of replication for both replicons has been centered on the graph at position 0; therefore, the plots start at 950 kb on Ch1 and 550 kb on Ch2. (**B**) AT-rich nature of MucR peaks determined by MEME analysis of the top 150 MucR peaks identified in the *B. abortus* 2308 genome by ChIP-seq. AT-rich sequence shown is present in 71/150 peaks.

To better define the extent of the *Brucella* MucR regulon*,* identify direct targets of this regulator, and determine the general characteristics of MucR binding sites across the *B. abortus* 2308 genome, we performed ChIP-seq on this strain and the isogenic *mucR* mutant CC092. We identified 546 MucR ChIP-seq peaks across the genome of the parental strain (Fig. 2A). These peaks are evenly distributed between both chromosomes with 352 peaks on Chromosome 1 (Ch1) and 194 peaks on Chromosome 2 (Ch2) equating to 1 peak per ∼ 6 kb across both chromosomes (Table S1). The average peak width was 584 bp. The average AT content of all peaks was 49.2%, which is higher than the average AT content across the entire *B. abortus* 2308 genome (42%). For the top 150 peaks, the average AT content was even higher (53.6%). To determine if there is a consensus binding sequence for MucR in the *B. abortus* 2308 genome, we employed the set of web-based bioinformatics tools known as the MEME Suite (https://meme-suite.org/meme/) (51) to analyze the sequences of the top 150 MucR enrichment sites (see Materials and Methods). We did not identify a highly conserved sequence but found a degenerate 26 bp AT-rich sequence in 71 of the peaks (Fig. 2B). Remarkably, of the 61 MucR-repressed genes reported in a previous microarray analysis (34), we found 55 (i.e., 91%) with a ChIP-seq peak at their confirmed or predicted promoter regions (Table 1). These findings provide further evidence that MucR functions as an H-NS-like gene silencer on a global scale in *Brucella*.

**TABLE 1:** MucR ChIP-seq peaks detected upstream of genes defined as being in the MucR regulon by microarray analysis in *B. abortus* 2308

Gene	Annotation	Description/proposed function	Effect of MucR on gene expression in *B. abortus* 2308^*a*^	MucR ChIP peak(s)^*e*^
Outer membrane/surface proteins				
*btaE*^*b*^^,*c*^	BAB1_0069	Autotransporter adhesin	–	37,177
*omp25d*^*b*^^,*c*^	BAB1_0115	Outer membrane protein	–	59
	BAB1_1489	Uncharacterized outer membrane protein	–	52
*btaF*^*b*^^,*c*^	BAB1_1854	Autotransporter adhesin	–	91
*bigB*^*b*^^,*c*^	BAB1_2012	Autotransporter adhesin	–	82
*bmaC*^*b*^^,*c*^	BAB2_1107	Autotransporter adhesin	–	5,394, 526
Genetic regulators				
*babR*^*b*^	BAB1_0190	LuxR-type quorum-sensing regulator	–	35
*glpR*	BAB1_0198	DeoR-type transcriptional regulator	–	140
*bpdB*^*b*^^,*c*^	BAB1_0512	c-di-GMP-specific phosphodiesterase	–	31
*mucR*^*b*^^,*c*^	BAB1_0594	H-NS-like protein	–	57
*nolR*^*b*^^,*c*^	BAB1_1605	ArsR-type transcriptional regulator	+	53
*deoR1*^*b*^^,*c*^	BAB2_0143	DeoR-type transcriptional regulator	−	ND^*d*^
	BAB2_0806	LuxR-type transcriptional regulator	–	1
	BAB2_0807	Crp-type transcriptional regulator	–	1
*iclR*	BAB2_0873	Crp family transcriptional regulator	–	58
Transporters				
*lldP*	BAB1_0738	L-lactate permease	+	ND
*rhaT*	BAB1_1893	DMT family membrane transporter	–	ND
*yhhQ*	BAB1_2015	Queuosine transporter	+	ND
*ansP*	BAB2_0055	Amino acid permease	–	98
*mfs*	BAB2_0348	MFS family transporter	+	214
*ftrA*^*b*^^,*c*^	BAB2_0840	Fe^2+^ transporter	+	ND
*ftrB*^*b*^^,*c*^	BAB2_0839	Fe^2+^ transporter	+	ND
*ftrC*^*b*^^,*c*^	BAB2_0838	Fe^2+^ transporter	+	ND
*ftrD*^*b*^^,*c*^	BAB2_0837	Fe^2+^ transporter	+	ND
Polysaccharide biosynthesis and modification genes				
*bcsA*	BAB1_0326	Exopolysaccharide biosynthesis	–	86
*manB*^*b*^^,*c*^	BAB1_0560	LPS O-chain biosynthesis	–	74,170
*exsB*	BAB1_1973	Exopolysaccharide biosynthesis	+	ND
*exsC*	BAB1_1974	Exopolysaccharide biosynthesis	+	ND
*exsD*	BAB1_1975	Exopolysaccharide biosynthesis	+	ND
*gtrA*	BAB2_0132	Polysaccharide biosynthesis/modification	−	64
*wcaA*	BAB2_0133	Polysaccharide biosynthesis/modification	−	64
*chbG*	BAB2_0134	Polysaccharide biosynthesis/modification	−	64
*arnT*	BAB2_0135	Polysaccharide biosynthesis/modification	−	64
Phage and transposon genes				
*tnsA*	BAB1_0746	Transposase-associated endonuclease	–	28,343
*rve*	BAB1_0747	Transposase-associated integrase	–	28,343
*tniQ*	BAB1_0750	Transposase	–	5,581
	BAB1_0751	Conserved hypothetical protein	–	2,281
Cellular metabolism and physiology genes				
*ialB*	BAB1_0070	Invasion-associated locus B protein	–	33,177
	BAB1_0087	DUF2794 family protein	–	72
*yeaQ*	BAB1_0459	Uncharacterized stress response protein	–	57
	BAB1_0745	Xre domain DNA-binding protein	–	28
*hppA*	BAB1_0793	Pyrophosphate-dependent proton pump	–	ND
*yebE*	BAB1_0893	Uncharacterized membrane protein	–	ND
*rssA*	BAB1_1099	Patatin family protein	–	115
*queFC*^*c*^	BAB1_1206	Queuosine biosynthesis	+	ND
*acm*	BAB1_1465	Muramidase	–	66,348
*mutT*	BAB1_1511	DNA mismatch repair	–	ND
*yraI*	BAB1_1529	SH3 domain protein	–	34
	BAB1_1689	DUF6536 family protein	–	417
*aqpZ*^*b*^	BAB1_2001	Aquaporin Z	–	79
*palA*^*b*^^,*c*^	BAB1_2011	Autotransporter assembly	−	158
	BAB1_2041	DUF4354 family protein	–	6
*rlpA*	BAB1_2138	Septal ring lytic transglycosylase	+	36
*cytB*	BAB2_0196	Cytochrome b561	–	41
*ampC*	BAB2_0257	Beta-lactamase	–	43
*ytcJ*	BAB2_0607	Metal dependent amidohydrolase	–	50
*dadA*	BAB2_0613	Amino acid oxidase	–	62
*dsbB*	BAB2_0846	Disulfide bond formation	–	102
*mazF*	BAB2_1072	Toxin/antitoxin system toxin	–	40
Genes encoding hypothetical proteins				
	BAB1_0013	Conserved hypothetical protein	–	10
	BAB1_0265	Conserved hypothetical protein	–	125
	BAB1_0324	Conserved hypothetical protein	–	60
	BAB1_0655	Conserved hypothetical protein	–	42
	BAB1_1035	Conserved hypothetical protein	–	51
	BAB1_1352	Conserved hypothetical protein	–	ND
	BAB1_1398	Conserved hypothetical protein	+	ND
	BAB1_1487	Conserved hypothetical protein	–	363
	BAB1_1509	Conserved hypothetical protein	–	19
	BAB1_1535	Conserved hypothetical protein	–	38
	BAB1_1536	Conserved hypothetical protein	–	38
	BAB1_2010	Conserved hypothetical protein	–	158
	BAB1_2021	Conserved hypothetical protein	–	5,675
	BAB2_0258	Conserved hypothetical protein	–	43
	BAB2_0450^c^	Conserved hypothetical protein	–	76
Genes that are no longer annotated or annotated as pseudogenes in *B. abortus* 2308				
	BAB1_0043	No longer annotated	–	106
	BAB1_0189	No longer annotated	–	35
*abiH*	BAB1_0271	Bacteriophage pseudogene	**–**	4
	BAB1_0554	Transposase pseudogene	–	32
	BAB1_0555	Transposase pseudogene	–	32
	BAB1_1125	No longer annotated	–	11
	BAB1_1604	No longer annotated	–	53
	BAB1_2000	No longer annotated	–	79
	BAB1_2002	No longer annotated	–	224
	BAB2_0092	No longer annotated	–	29
	BAB2_0861	No longer annotated	–	111
*gadB*	BAB2_0865	Glutamate decarboxylase pseudogene	–	15
*gadB*	BAB2_0866	Glutamate decarboxylase pseudogene	–	15
	BAB2_0867	No longer annotated	–	15
	BAB2_0887	No longer annotated	–	3
	BAB2_1151	No longer annotated	+	187

^*a*^ − indicates gene displayed greater than twofold increase in expression in *mucR* mutant vs parent, and + indicates gene displayed greater than twofold reduced expression in *mucR* mutant vs parent in microarray analysis (34).

^*b*^ Gene function has been experimentally validated in *Brucella.*

^*c*^ Gene been experimentally linked to virulence in *Brucella.*

^*d*^ ND, MucR ChIP peak not detected.

^*e*^ Peak numbers correspond to Table S1.

### Evidence that MucR/counter-silencer pairs regulate virulence gene expression in *Brucella*

**Fig 3:**
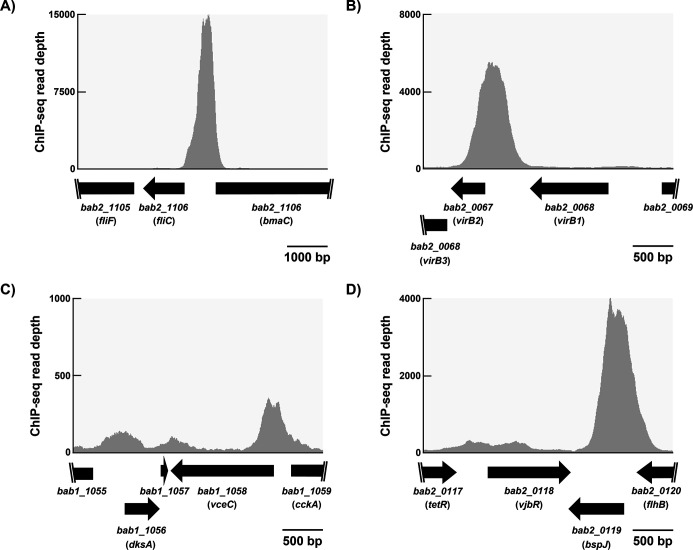
MucR ChIP-seq peaks are associated with genes encoding known *B. abortus* 2308 virulence determinants including the autotransporter BmaC (**A**), the T4SS (**B**), T4SS effectors (**C and D**), and VjbR (**D**). ChIP-seq read depth is shown above annotated open reading frames (ORFs) within each region. Scale bars of the x-axis are shown below each graph.

Of the targets of MucR repression, which have MucR ChIP-seq peaks associated with their promoters, seven genes (*btaE*, *btaF*, *bmaC* [Fig. 3A], *bigB*, *omp25d*, *bpdB*, and *manB*) play well-documented roles in *Brucella* virulence (52–59). In addition, our ChIP-seq analysis identified MucR ChIP-seq peaks in the promoter regions of other important *Brucella* virulence genes including those encoding the Type IV secretion system (T4SS) (Fig. 3B), T4SS effectors (Fig. 3C and D), the quorum-sensing regulator VjbR (Fig. 3D), and the LPS O-chain biosynthetic pathway (Table 2). It remains to be experimentally determined to what extent MucR affects the expression of these latter genes. Nonetheless, these results suggest that similar to H-NS and the H-NS-like gene silencers in other bacterial pathogens, MucR’s contribution to virulence in *Brucella* may be through preventing the gratuitous expression of virulence genes when the corresponding gene products provide no fitness benefit during the infectious life cycle.

**TABLE 2:** *Brucella* virulence-associated genes with MucR ChIP-seq peaks in *B. abortus* 2308 for which regulatory links to MucR have not been experimentally established

Gene	Annotation	Proposed function	MucR ChIP peak(s)^*a*^
Outer membrane/surface proteins			
*omp31b*	BAB1_1639		250,198
*bigA*	BAB1_2009	Inverse autotransporter adhesin—attachment to mammalian cells	9,570
Type 4 secretion machinery and effectors			
*virB1*	BAB2_0068	T4SS assembly and function	476
*virB2*	BAB2_0067	T4SS assembly and function	71
*virB4*	BAB2_0065	T4SS assembly and function	225,209
*virB6*	BAB2_0063	T4SS assembly and function	315
*sagA*	BAB1_1002	T4SS assembly and function	17
*vceC*	BAB1_1058	T4SS effector—modulates host immune response	232
*btpA*	BAB1_0279	T4SS effector—modulates host immune response	166
*btpB*	BAB1_ 0756	T4SS effector—modulates host immune response	524
*bspA*	BAB1_0678	T4SS effector—inhibits host ER-associated degradation pathway	461
*bspE*	BAB1_1675	T4SS effector—function unknown	223
*bspF*	BAB1_1948	T4SS effector—inhibits vesicular transport in host cells	164
*bspJ*	BAB2_0119	T4SS effector—inhibits host cell apoptosis	89
*bpe275*	BAB1_1275	T4SS effector—function unknown	482
LPS biosynthesis			
*pgm*	BAB1_0055	Core biosynthesis	278
*wbkD*	BAB1_0534	O-chain synthesis	488
*wbkF*	BAB1_0535	O-chain synthesis	318
*wbkC*	BAB1_0540	O-chain synthesis	6,590
*wzt*/*rfbB*	BAB1_0542	O-chain synthesis	47
*wzm*/*rfbD*	BAB1_0543	O-chain synthesis	47
*per*	BAB1_0544	O-chain synthesis	47
*gmd*	BAB1_0545	O-chain synthesis	118
*wbkA*	BAB1_0553	O-chain synthesis	32
*manC*	BAB1_0561	O-chain synthesis	74,170,229
*manA*	BAB1_0562	O-chain synthesis	74,170,229
*wbkE*	BAB1_0563	O-chain synthesis	116
*wadA*	BAB1_0639	Core biosynthesis	341,322
*wadD*	BAB1_0953	Core biosynthesis	60
*wboA*	BAB1_0999	O-chain synthesis	1,217
*wboB*	BAB1_1000	O-chain synthesis	1,217
Transcriptional regulators			
*otpR*	BAB1_2006	OmpR-type transcriptional regulator	241
*vjbR*	BAB2_0118	LuxR-type quorum-sensing regulator	242
*lovhK*	BAB2_0652	Histidine kinase	212

^*a*^ Peak numbers correspond to Table S1.

Importantly, multiple *Brucella* putative promoters with MucR ChIP-seq peaks also have experimentally verified binding sites for transcriptional activators, such as MdrA (49, 60), HutC (5, 49), VjbR (61, 62), BvrR (4, 63), or CtrA (64), which overlap with the MucR peaks (Fig. 1A; Table S2). These results further support that MucR functions as an H-NS-like gene silencer and that these latter transcriptional activators may serve as MucR ‘counter-silencers’.

**Fig 4:**
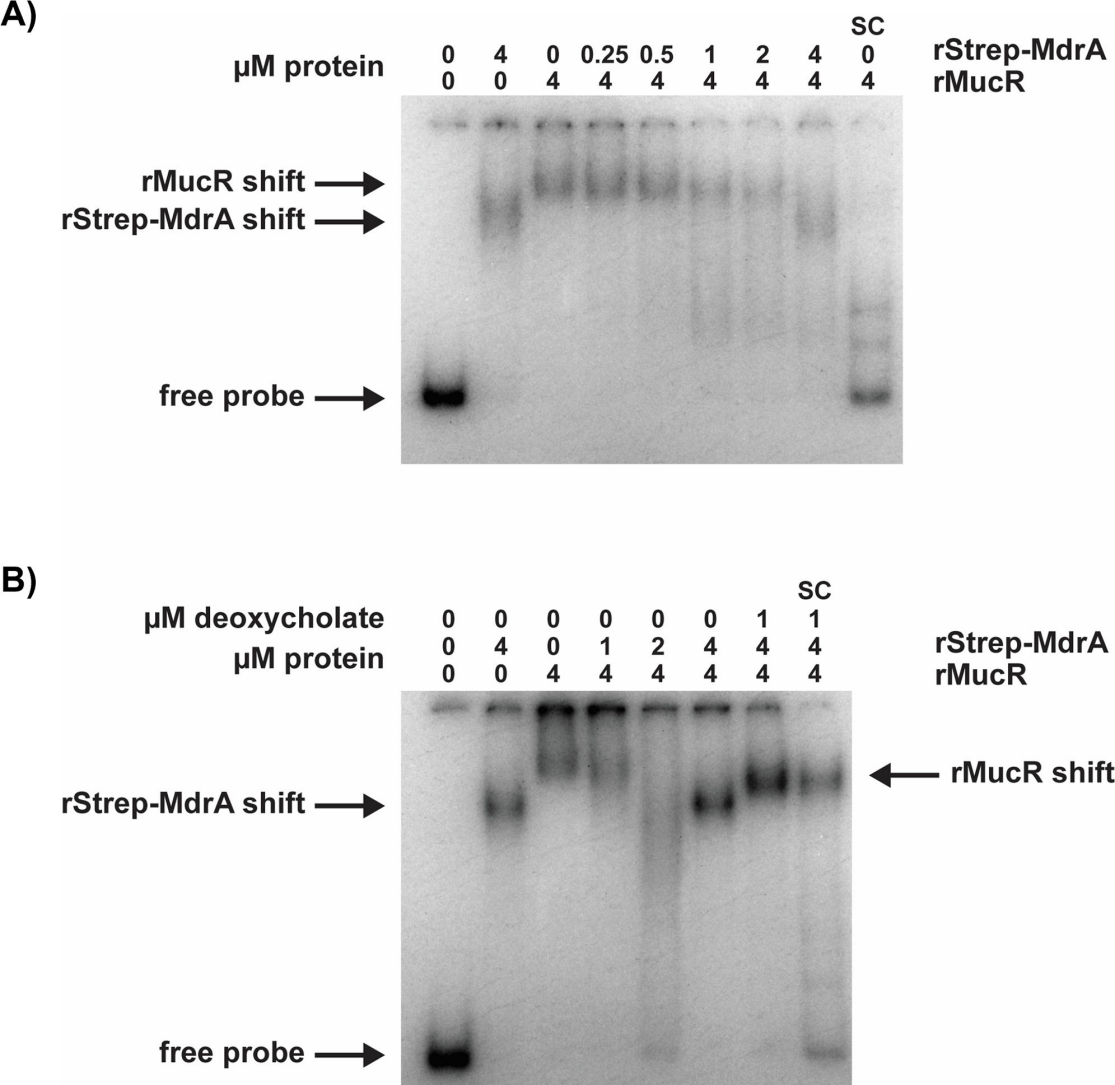
(**A**) Competitive displacement of MucR from the *B. abortus* 2308 *btaE* promoter by the MarR-type transcriptional activator MdrA in an EMSA. (**B**) Deoxycholate inhibits the ability of MdrA to displace MucR from the *btaE* promoter. SC denotes the addition of unlabeled specific competitor DNA. A 192 bp *btaE* promoter fragment (F3/R3, Table S4) was used in both panels A and B.

The presence of overlapping binding sites for the MarR-type transcriptional activator MdrA and MucR in the *btaE* promoter (Fig. 1A) provided us with an opportunity to test the hypothesis that these two regulators form a silencer/counter-silencer pair in *Brucella*. To examine this possibility, we assessed the capacity of MdrA to compete with MucR for binding to the *btaE* promoter in an EMSA. As shown in Fig. 4A, MdrA can displace MucR from the *btaE* promoter in a dose-dependent fashion. Importantly, MdrA can no longer displace MucR from the *btaE* promoter when deoxycholate, a regulatory co-factor that inhibits MdrA’s DNA-binding activity (60), is included in the reaction mixture (Fig. 4B). These results suggest that MucR and MdrA represent a functional gene silencer/counter-silencer pair in *Brucella* and that the counter-silencing function of MdrA can be modulated by an external stimulus.

### Evidence that MucR serves as a xenogeneic silencer in *Brucella*

**Fig 5:**
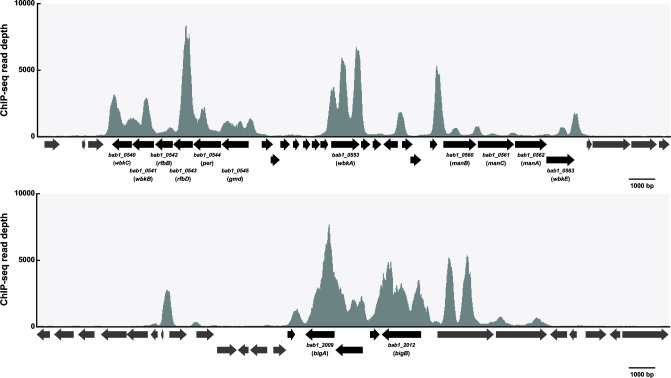
MucR ChIP-seq peaks extend across Region 16 (top) and Core 8 (bottom) genomic islands described by Wattam et al. (65). ORFs falling within/without the described genomic islands are shown in black/gray, respectively. Select gene names are shown. ChIP-seq read depth is shown above annotated ORFs within each region. Scale bars of the x-axis are shown below each graph.

The fact that the classical *Brucella* spp. reside predominantly in close association with their mammalian hosts and inhabit an intracellular niche is thought to limit their opportunities for HGT with other bacteria (65). Nevertheless, there are compelling data generated from comparative genomic studies indicating that the acquisition of certain virulence genes such as those involved in the biosynthesis of the perosamine O-chain of the LPS and the T4SS played an important role in the evolution of these bacteria as pathogens (65). Interestingly, our ChIP-seq analysis identified multiple and often contiguous MucR peaks in 26 of 38 genomic islands described in *B. abortus* 2308 (65–67) (Table 3). The contiguous peaks detected in the genomic islands described as Region 16 (Fig. 5, top) and Core 8 (Fig. 5, bottom) by Wattam et al. (65), which encode LPS O-chain biosynthesis genes and the autotransporter adhesin BigB, respectively, are particularly notable in this regard. Our findings indicate that the function of the *Brucella* MucR as a xenogeneic silencer warrants a more detailed investigation.

**TABLE 3:** MucR ChIP-seq peaks in the genomic islands described in *B. abortus* 2308

Genomic island	Chromosomal location	MucR ChIP peak(s)^*e*^
GI-1^*a*^	BAB1_1101-1106	2,754
GI-2^*a*^	BAB1_0983-1007	71,217,501,536
GI-3^*a*^	BAB1_0249-0279	424,125,166,251,287
GI-4^*a*^	BAB1_0020-0022	33,442
GI-5^*a*^	BAB2_1033-1073	40,173,207,235,270,451,473,491
GI-6^*a*^	BAB2_0682-0686	404
GI-7^*a*^	BAB2_0781-0786	1,133
GI-9^*a*^	BAB2_0830-0832	ND^*d*^
Region 1^*b*^	BAB1_0608-0623	ND
Region 2^*b*^	BAB1_1079-1084	432,519
Region 3^*b*^	BAB1_1376-1391	ND
Region 4^*b*^	BAB1_1636-1641	198,230,239,250
Region 5^*b*^	BAB2_0012-0016	ND
Region 6^*b*^	BAB2_0034-0041	ND
Region 7^*b*^	BAB2_0131-0134	64
Region 8^*b*^	BAB2_0214-0228	286
Region 9^*b*^	BAB2_0857-0865	23,111,113
Region 10^*b*^	BAB2_0808-0820	119
Region 11	BAB2_0617-0624	154,155,203,225,259
Region 12^*b*^	BAB2_0432-0442	188,213,349
Region 13^*b*^	BAB2_1152-1162	451,491
Region 14^*b*^	BAB2_1113-1135	6,873,159,472
Region 15^*b*^	BAB2_0057-0068	71,209,315,425,476
Region 16^*b*^	BAB1_0540-0563	32,477,490,114,116,118,170,229,304
Core 1^*b*^	BAB1_0288-0293	25,301
Core 2^*b*^	BAB1_0740-0756	22,285,581,128,202,231,281,343,524
Core 3^*b*^	BAB1_0814-0819	45
Core 6^*b*^	BAB1_1490-1493	ND
Core 7^*b*^	BAB1_1860-1862	215,387
Core 8^*b*^	BAB1_2008-2012	707,882,158
Core 10^*b*^	BAB2_0392-0403	186,365,470,486
GI03^*c*^	BAB1_0294-0296	ND
GI05^*c*^	BAB1_0124-0128	ND
GI07^*c*^	BAB2_0382-0387	ND
GI08^*c*^	BAB2_0588-0605	ND
GI09^*c*^	BAB2_0599-0605	123,236
GI14^*c*^	BAB1_0612-0616	ND
GI15^*c*^	BAB1_0606-0613	ND

^*a*^ Rajashekara et al. (66).

^*b*^ Wattam et al. (65).

^*c*^ Zhong et al. (67).

^*d*^ ND = not detected.

^*e*^ Peak numbers correspond to Table S1.

### MucR contributes to the organization of both chromosomes in *B. abortus* 2308

The same biochemical properties that allow H-NS and H-NS-like proteins to be effective gene silencers also allow them to play integral roles in nucleoid compaction in bacterial cells (20, 27, 68). This raised an obvious question. Is MucR performing a nucleoid structuring function in *Brucella*? Chromosomal conformation capture (Hi-C) is an approach that quantifies genome-wide DNA-DNA interactions (69). It employs formaldehyde cross-linking to capture chromosomal loci that lie in proximity to each other and applies restriction digestion, ligation, and paired-end sequencing to identify these interacting loci. The data obtained are then used to construct positional maps portraying the interactions of these loci within and between chromosomes. Hi-C has been used to study the genome structure in multiple bacteria (70–76), and more importantly, it has also been used to demonstrate that H-NS plays a direct role in structuring the bacterial nucleoid (76). Specifically, in *E. coli*, the absence of H-NS increases short-range DNA contacts, suggesting that in wild-type cells H-NS prevents H-NS binding sites from interacting with neighboring loci (76).

**Fig 6:**
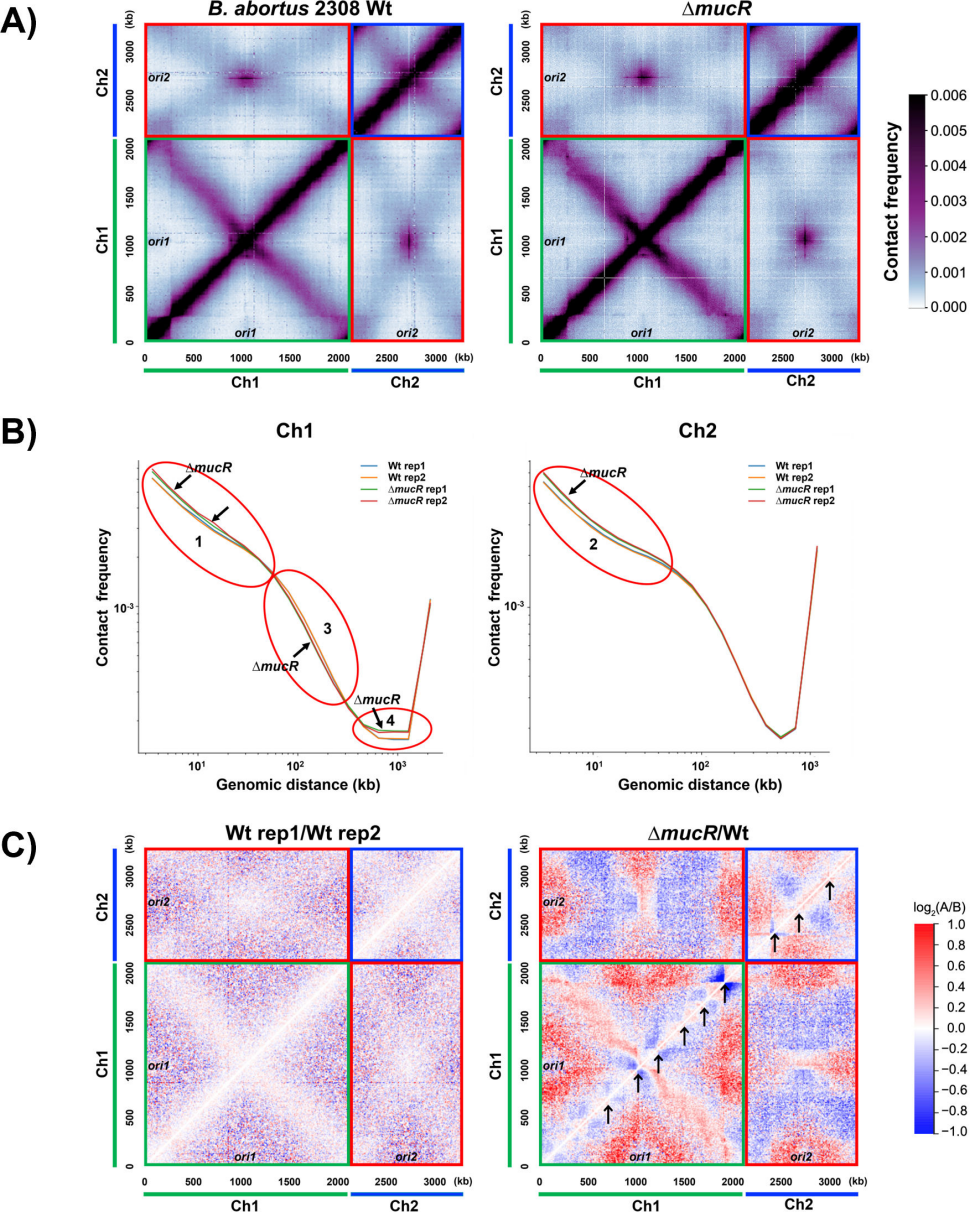
(**A**) Normalized Hi-C contact frequency maps of the genomes of *B. abortus* 2308 (left) and an isogenic *mucR* mutant (right). The x-and-y axes correspond to the genome position in kb. The data are plotted in 5 kb bins. Chromosome 1 (Ch1, green bar), Chromosome 2 (Ch2, blue bar), and their respective origins (*ori1*, *ori2*) are labeled. Origins have been situated to the middle of each replicon for better visualization of the interactions within these regions. The starting position for Ch1 is 950 kb and for Ch2 is 550 kb, but the Hi-C axes are shown as a contiguous number to represent the distance in kb. The scale bar for Hi-C interaction scores (contact frequency) is shown on the right. The Hi-C map can be divided into four parts: a Ch1 interaction map in the bottom left quadrant (green box), a Ch2 interaction map in the top right quadrant (blue box), and a Ch1-Ch2 interaction map with identical, mirrored copies in the top left and bottom right quadrants (red boxes). The strong primary diagonal lines on the map from bottom left to top right demonstrate the short-range interactions along the chromosomes. The weak secondary diagonal lines extending from top left to bottom right demonstrate interactions between loci on opposite arms of each chromosome. (**B**) Hi-C contact probability decay curves show the averaged contact frequency between every pair of loci on the chromosome separated by set distances. The x-axis indicates the genomic distance of separation in kb. The y-axis represents the averaged contact frequency in a logarithmic scale. The data are generated using 1 kb resolution. The red ovals (labeled 1–4) indicate differences between 2308 and the *mucR* mutant. Two biological replicates of each strain are shown. (**C**) Log_2_ ratio plots comparing different Hi-C matrices. Log_2_(matrix A/matrix B) was calculated and plotted in the heatmaps. The ratio of two biological replicates (Wt rep1/Wt rep2) is shown on the left and the ratio of *∆mucR* to Wt (*∆mucR*/Wt) is on the right. The color scale is shown. Black arrows point to a few examples of strong signatures that overlap with MucR ChIP-seq peaks that had fold enrichment greater than 10.

To determine the global structure of the *Brucella* genome, we performed Hi-C on *B. abortus* 2308 (Fig. 6A, left panel). Since *B. abortus* has two chromosomes, the genome-wide Hi-C interaction map can be divided into four distinct regions: a Ch1 interaction map in the bottom left quadrant, a Ch2 interaction map in the top right quadrant, and a Ch1-Ch2 interaction map with identical, mirrored copies in the top left and bottom right quadrants. Within each chromosome, strong short-range interactions are observed on the primary diagonal. When the Hi-C map was plotted in a different color scale, chromosome interaction domains were observed on both chromosomes (Fig. S2). Within Ch1, inter-arm interactions were evident by the secondary diagonal on the Hi-C map (Fig. 6A, left panel, bottom left quadrant), similar to what has been observed for many bacteria species (71–75). However, inter-arm interactions on Ch2 appeared weaker and less defined. Finally, between the chromosomes, we found strong interactions between the origin regions of the two chromosomes (purple area in the top left quadrant of Fig. 6A), and weak interactions along the replication arms of the two chromosomes (light blue X-shaped patterns in the top left quadrant of Fig. 6A). These inter-chromosomal interactions are similar to those observed for *Agrobacterium tumefaciens (*71). Overall, our WT Hi-C data are similar to the pattern reported in a different *Brucella* species, *Brucella melitensis*, in a recent study (77).

To determine if MucR plays a role in structuring the *Brucella* genome, we performed Hi-C in the isogenic *mucR* mutant CC092 (Fig. 6A, right panel) and compared the results with 2308 (Wt). To quantify the effect of MucR on genome organization, we plotted probability decay curves [Pc(s)] for the two strains (Fig. 6B). Pc(s) curves analyze the averaged contact frequency between all pairs of loci on the chromosome separated by set distance(s). As shown in Fig. 6B, biological replicates of each strain had overlapping Pc(s) curves, demonstrating the reproducibility and robustness of the Hi-C assay. Importantly, in the absence of MucR, short-range interactions (<50 kb) on both chromosomes increased (Fig. 6B, red ovals 1 and 2), indicating that MucR binding to DNA prevents these regions from interacting with other nearby regions. This effect is the same as the effect of H-NS on the *E. coli* chromosome (76). These results support the idea that MucR has a nucleoid-structuring role like the *E. coli* H-NS protein.

Since *Brucella* has two chromosomes, this also allowed us to investigate whether MucR affects the two chromosomes differently and whether it affects inter-chromosomal interactions. Indeed, we found that MucR affected Ch1 and Ch2 differently—although increasing short-range interactions was the only change of *∆mucR* on Ch2, for Ch1, the absence of MucR was also correlated with a slight decrease in mid-range (50–400 kb) interactions (Fig. 6B, oval 3) and a prominent increase in long-range (400–1000 kb) interactions (Fig. 6B, red oval 4).

To understand these changes in Pc(s) curves in the context of Hi-C interactions, we plotted the ratio of *∆mucR* Hi-C map to that of Wt (Fig. 6C). As a control, we plotted the ratio of two biological replicates of the Wt Hi-C maps (Fig. 6C, left panel), which mostly showed white pixels indicating no change, or a mix of blue and red pixels indicating the “noise” between biological replicates (Fig. 6C, left panel). The ratio between the two biological replicates of *∆mucR* looked very similar (Fig. S3). Strikingly, in the ratio between *∆mucR* and Wt, we saw large contiguous regions of blue or red pixels, indicating that genome interactions have dramatically changed in *∆mucR*. Specifically, along the primary diagonals of both chromosomes, there were mostly red pixels, indicating an increase in short-range interactions in ∆*mucR*, consistent with the Pc(s) analyses (Fig. 6B, ovals 1 and 2). In addition, along the primary diagonals, there were some dramatic changes in the domain boundaries. Importantly, most of the affected domain boundaries overlapped with MucR ChIP-seq peaks that had greater than 10-fold enrichment (Fig. 6C, right, black arrows), suggesting that these changes correlated with MucR binding to these sites.

On Ch1, we saw red regions at inter-arm interactions and at the periphery of the map outside of the diagonals (Fig. 6C, right, red areas in green box), showing an increase in long-range DNA contacts, which is consistent with Pc(s) analyses (Fig. 6B, oval 4). On Ch2, the blue regions indicated reduced inter-arm interactions in *∆mucR*, and the red regions indicated an increase of other long-range interactions (Fig. 6C, right, blue and red areas in blue box). Interestingly, after averaging, the overall long-range DNA contacts in Ch2 seemed to be the same between Wt and ∆*mucR* (Fig. 6B, right panel). Finally, MucR also seemed to affect the interactions between the two chromosomes (Fig. 6C, right, blue and red areas in red boxes). Specifically, we saw a great decrease (i.e., blue areas) of the X-shaped interactions observed on the *Brucella* Hi-C map and an increase (i.e., red areas) outside of the X-shaped region (Fig. 6A, red boxes and Fig. 6C, right panel red boxes), which represented reduced alignment between the arms of Ch1 and Ch2, and increased Ch1-Ch2 contacts other than arm alignment.

Although the exact molecular mechanism is currently unknown, it is clear from our experiments that MucR causes both local and global changes in genome structure, both within chromosomes and between chromosomes, to an extent much greater than what was observed for *E. coli* H-NS (76). Therefore, MucR plays a role in organizing the overall structure of the *Brucella* genome, consistent with the proposition that MucR represents a novel type of H-NS-like protein (41, 42).

### *E. coli hns* rescues the distinctive growth defect and elevated *btaE* expression exhibited by a *B. abortus mucR* mutant

**Fig 7:**
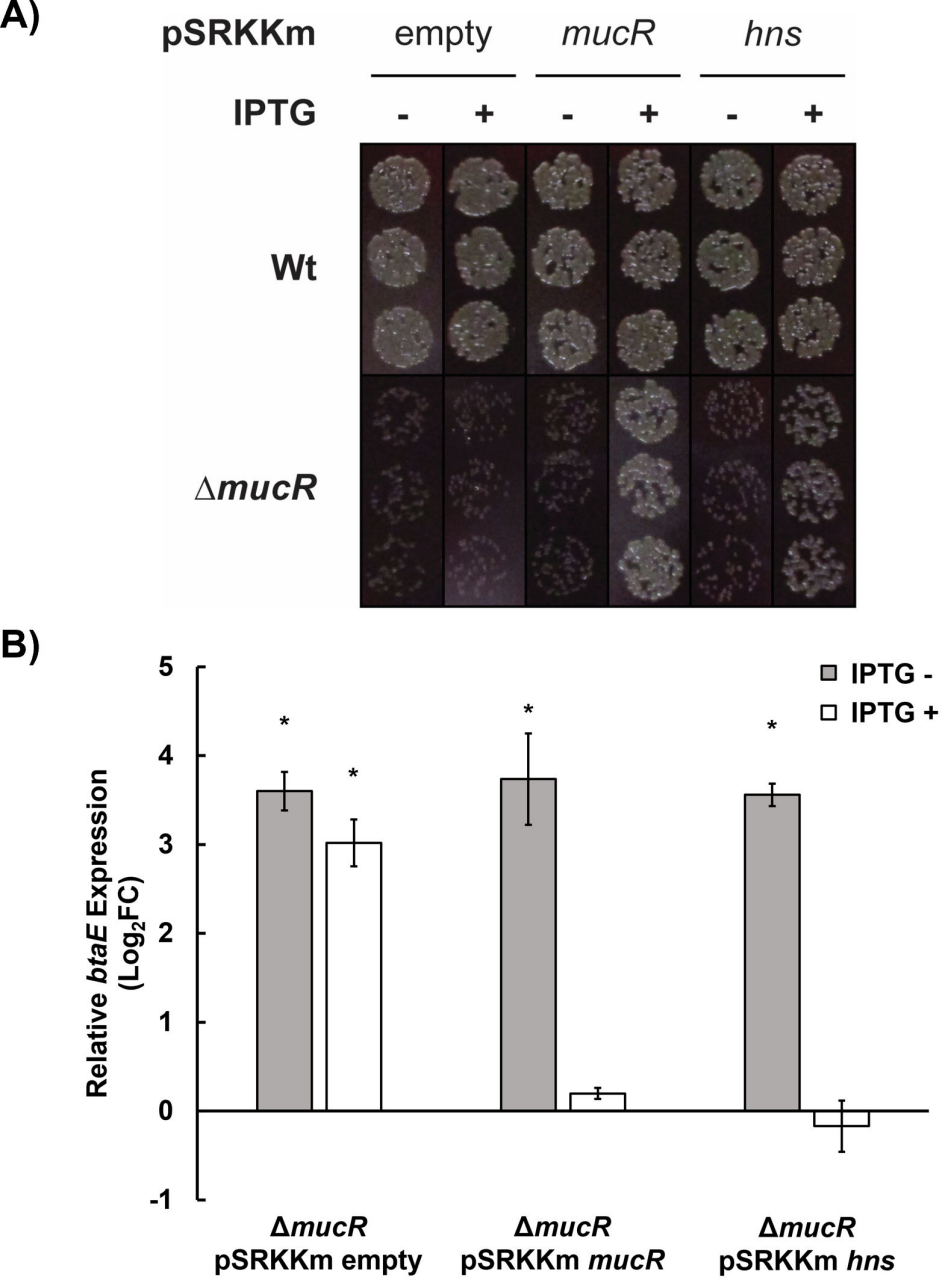
(**A**) Plasmid-borne *E. coli hns* can complement the growth defect of *B. abortus* 2308 *mucR* mutant. *B. abortus* 2308 (above) and an isogenic Δ*mucR* mutant (below) carrying pSRKKm with *mucR*, *hns*, or no insert (empty) were grown and spotted onto SBA with (+) or without (−) IPTG (see Materials and Methods). (**B**) Plasmid-borne expression of *E. coli hns* can complement elevated *btaE* expression of *B. abortus* 2308 Δ*mucR*. RNA was isolated from *B. abortus* Δ*mucR* carrying either pSRKKm alone (empty, left), pSRKKm *mucR* (middle), or pSRKKm *hns* (right) grown in the presence (+) or absence (−) of IPTG and used for RT-PCR (see Materials and Methods). Relative expression is shown as a Log2 fold-change for each treatment relative to Wt *B. abortus* carrying empty pSRKKm. * indicates a significant difference from Wt (one sample *t*-test; *P*-value < 0.05).

The *B. abortus mucR* mutant CC092 has a distinctive slow growth characteristic, displaying an ~24 h delay in the formation of visible colonies on Schaedler blood agar plates compared to the parental 2308 strain and this phenotype can be rescued by a plasmid-borne copy of the wild-type *mucR* (34) (Fig. 7A). We have reliably used this phenotype in genetic screens to assess the functionality of mutated *Brucella mucR* alleles with site-directed mutations and evaluate the capacity of *mucR* genes from other α-proteobacteria to function in *Brucella* (36). Remarkably, a plasmid-borne copy of the *E. coli hns* gene rescued the characteristic growth defect of the *B. abortus mucR* mutant (Fig. 7A). Since the ability of the *E. coli hns* gene to rescue a distinctive mutant phenotype has been used to demonstrate the ‘functional homology’ between H-NS and the HNS-like proteins in other bacteria (23), this result provides further evidence that MucR is in fact a novel-type of H-NS-like protein. Additionally, a plasmid-borne copy of the *E. coli hns* gene was able to reduce the elevated *btaE* expression of a *B. abortus mucR* mutant (Fig. 7B) suggesting that *hns* can also rescue elevated transcription of certain MucR targets in the absence of *mucR*.

## DISCUSSION

Silencing/counter-silencing is a hallmark of gene regulation by H-NS and H-NS-like proteins, where gene silencing by H-NS or H-NS-like proteins is relieved through a variety of mechanisms including topological changes in DNA (78) and/or competitive binding by antagonistic transcriptional activators (9, 12, 79), resulting in tight, temporal control of gene expression. In many pathogenic bacteria, H-NS-mediated silencer/counter-silencer interactions are critical for successful interactions with the host and evasion of the host immune system through regulation of many host-associated functions including cellular attachment (18, 80) and expression of secretion systems and effectors (81, 82), which are only needed at specific times during infection. In keeping with its proposed function as an H-NS-like gene silencer (40, 41), the data presented here suggest that MucR works in concert with antagonistic transcriptional activators, e.g., ‘counter-silencers’ to ensure the proper temporal expression of virulence genes in *Brucella*.

The presence of documented DNA-binding sites for the transcriptional activators VjbR, BvrR, and CtrA that overlap with MucR binding sites in the promoters of specific virulence genes is particularly striking because VjbR and BvrR are known to be responsive to host-specific environmental stimuli (4, 6). CtrA, on the other hand, is the master regulator of cell cycle genes and experimental evidence indicates that *Brucella* cells in G1 phase of the cell cycle are more infectious for mammalian cells than *Brucella* cells in other stages (83). The virulence genes that appear to be coordinately regulated by MucR and VjbR, BvrR, and/or CtrA (Table S2) are also interesting because the corresponding gene products would be expected to be required during different stages of the infectious process. Experimental evidence indicates that the polar autotransporter adhesins BtaE and BmaC, for instance, are important during early stages of infection, specifically attachment to, and entry into, epithelial cells (49, 54). The corresponding genes also appear to be very tightly regulated since only 1%–4% of the bacterial cells in a culture express the genes during *in vitro* growth. The genes encoding the T4SS and its effectors, in contrast, are required for proper trafficking of the *Brucella* containing vacuoles in host cells, and it is well-documented that BvrR and VjbR work in concert to induce the expression of the genes encoding the T4SS in response to environmental conditions encountered in the intracellular environment. It is also interesting to note that the data presented here suggest that MucR and BvrR may coordinate the proper temporal expression of *vjbR*. Additionally, the fact that the MarR-type regulator, MdrA, can displace MucR from the *btaE* promoter suggests that MdrA may be an important counter-silencer for select MucR targets. MdrA has been shown previously to be required for attachment to epithelial cells and regulates both the *btaE* and *virB* promoters in conjunction with other transcriptional regulators, including IHF and HutC (49, 60). Experiments are underway to determine how MucR and these proposed counter-silencers work together to coordinate expression of the AT adhesin, T4SS, and T4SS effector genes, and to determine how important the competing activities of the MucR/counter-silencer pairs on individual virulence genes are for the wild-type virulence of *Brucella*.

Recent work has supported a role for MucR from *Sinorhizobium* as a xenogeneic silencer (40, 42), which is another role attributed to H-NS and H-NS-like proteins. Xenogeneic silencing is thought to provide a mechanism for preventing detrimental, unregulated expression of horizontally acquired and/or foreign genes and allowing stable integration of acquired genes within the pre-existing genetic and transcriptional networks of the cell. The identification of MucR binding regions throughout most (26/38) of the genomic islands proposed within the *B. abortus* 2308 genome (Table 3) supports a conserved role for MucR as an H-NS-like xenogeneic silencer in *Brucella*. The presence of many important virulence factors within proposed genomic islands, including the T4SS and LPS synthesis genes, and the presence of extended MucR binding regions across these loci (Fig. 5) suggest that xenogeneic silencing of these regions by MucR is important for host adaptation and virulence. However, future experiments will be needed to determine the precise role of MucR in the temporal regulation of gene expression within genomic islands and its significance to *Brucella*’s evolution as a mammalian pathogen (65).

In addition to their role in gene silencing, H-NS and H-NS-like proteins also play a role in shaping the structure of the bacterial nucleoid through a combination of DNA bending, bridging, and stiffening activities. Here, we show that a *mucR* mutant has global changes in DNA contacts by Hi-C, which is to our knowledge the first time MucR has been implicated as a global nucleoid structuring protein. Specifically, on both chromosomes, MucR reduces short-range (<50 kb) DNA interactions, similar to what has been observed for *E. coli* H-NS (76). In addition, we show that MucR affects the two chromosomes differently, and it also alters inter-chromosomal contacts. Although the molecular mechanism is still lacking, these findings are consistent with the idea that MucR plays a role in globally structuring the *Brucella* genome.

In addition to H-NS, there are other nucleoid-associated proteins that contribute to chromosomal structuring and maintenance, including HU, IHF, Fis, Dps, and SMC. *Brucella* spp. also possess genes encoding HU, IHF, Dps, and SMC. Interestingly, while in many bacteria HU is essential and IHF is dispensable, the opposite is true in *Brucella*: IHF is essential, and HU is dispensable (84). This suggests that chromosomal maintenance in *Brucella* may have diverged from other bacteria. It will be interesting to dissect the contribution of MucR and other nucleoid-associated proteins to chromosomal maintenance in *Brucella* and define their roles in host-adaptation. It will also be imperative to determine if MucR can bend, bridge, and stiffen DNA strands and evaluate how these properties impact the capacity of this protein to both serve as a gene silencer and structure the nucleoid.

In total, the work presented here solidifies the role of MucR as an H-NS-like protein in *Brucella*. Future work will be needed to identify the molecular mechanisms underlying counter-silencing and the importance of temporal regulation of discrete targets by MucR during host association and pathogenesis. Our observation that *hns* from *E. coli* is capable of rescuing elevated *btaE* expression and the growth defect of a *Brucella mucR* mutant indicates that these corresponding proteins share a conserved function in the bacterial cell. This is intriguing considering the limited amino acid sequence conservation between H-NS and MucR and the differing functional oligomeric states demonstrated for these proteins (39, 40). Further comparison of *hns* complementation with respect to other virulence gene regulation and chromosomal maintenance in a *Brucella mucR* mutant will help us better understand the shared functions and molecular mechanisms of these two proteins.

## MATERIALS AND METHODS

### Bacterial strains and growth conditions

*B. abortus* 2308 and derivative strains were grown on Schaedler agar (Becton, Dickinson and Co., Sparks, MD, USA) supplemented with 5% defibrinated bovine blood (SBA) incubated at 37°C under 5% CO_2_ or in brucella broth (Becton, Dickinson, and Co., Sparks, MD, USA) incubated at 37°C with shaking. *Escherichia coli* strains were grown on Luria-Bertani (LB) agar at 37°C or in LB broth with shaking at 37°C. Growth media were supplemented with carbenicillin (100 µg/mL), ampicillin (100 µg/mL), or kanamycin (45 µg/mL) when appropriate.

### Recombinant plasmid construction

The *mdrA* coding region was amplified from *B. abortus* 2308 genomic DNA by PCR using Phusion high-fidelity DNA polymerase (New England Biolabs, Ipswich, MA, USA) with primers KP015 and KP016 (Table S4) and then cloned into pASK-IBA7+ (IBA, Göttingen, Germany) pre-digested with EcoRI and BamHI using NEBuilder HiFi DNA assembly (New England Biolabs, Ipswich, MA, USA) following manufacturer’s recommended conditions. The resulting plasmid, pIB314 (Table S3), encodes MdrA with an amino-terminal epitope tag (Strep-tag II). This construction was confirmed by restriction digest mapping and DNA sequence analysis (Eurofins Genomics, Louisville, KY, USA).

The *mucR* coding region was amplified from *B. abortus* 2308 genomic DNA by PCR employing primers IB311 and IB312 (Table S4) and then cloned into pTXB1 (New England Biolabs, Ipswich, MA, USA) pre-digested with NdeI and SapI using NEBuilder HiFi DNA assembly. The resulting plasmid, pIB315 (Table S4), encodes MucR fused to an intein-/chitin-binding domain and this construction was confirmed by restriction digest mapping and nucleotide sequence analysis.

The *mucR* gene from *B. abortus* 2308 and the *hns* gene from *E. coli* MG1655 were amplified by PCR from genomic DNA from the corresponding bacteria using primers IBP325 and IBP326 or IBP327 and IBP328, respectively (Table S4), using *Taq* polymerase (New England Biolabs, Ipswich, MA, USA). The resulting DNA fragments were then cloned into pGEM-T-Easy (Promega, Madison, WI, USA) before being subsequently excised and cloned into the unique NdeI and HindIII sites of pSRKKm (85). The resulting plasmids, pIB316 and pIB317, contained *mucR* and *hns* fused in-frame to the start codon of the *lacZ* gene of the parent plasmid, and these constructions were confirmed by restriction enzyme mapping and DNA sequence analysis.

### Purification of recombinant MucR and MdrA proteins

Recombinant versions of the *Brucella* MucR and MdrA containing an amino-terminal Strep-tag II were purified as described previously (34) with minor alterations. Briefly, *E. coli* BL21 containing prMucR or pIB314 (Table S3) was grown by shaking in LB at 37°C to an optical density at 600 nm (OD_600_) of ~0.6 before adding anhydrotetracycline (100 µg/mL, final concentration) to the culture to induce recombinant protein production. Following 4 h subsequent incubation at 37°C, bacterial cells were collected by centrifugation (5000 × *g* for 10 min at 4°C) and lysed by a combination of treatment with CelLytic B cell lysis reagent (Sigma-Aldrich, St. Louis, MO, USA) supplemented with phenylmethanesulfonyl fluoride (1 mM, final concentration) and 2× passage through a French pressure cell press at 1000 psi. Insoluble debris was pelleted by centrifugation (5000 × *g* for 10 min at 4°C) and the cleared lysate was passed through an affinity column packed with *Strep-*Tactin Sepharose (IBA, Göttingen, Germany). The column was washed with buffer W (100 mM Tris-HCl and 300 mM NaCl) until the optical density at 280 nm (OD_280_) was <0.001. The protein was then eluted from the column with 2.5 mM desthiobiotin in buffer W, and the concentration and purity of eluted proteins were determined by absorbance measurements at 280 nm (A_280_) with a NanoDrop spectrophotometer (Thermo Fisher Scientific, Waltham, MA, USA) and SDS-PAGE analysis.

Purification of rMucR expressed from pIB315 was performed using manufacturer-recommended procedures (IMPACT kit, New England Biolabs, Ipswich, MA, USA). *E. coli* BL21 carrying pIB315 was grown as above but induced for recombinant protein expression by adding 0.4 mM isopropyl-β-D-1-thiogalactopyranoside (IPTG). Bacterial cells were collected post-induction and cleared lysates were prepared as above and passed over an affinity column packed with chitin resin that had been equilibrated with 10 column volumes of column buffer (20 mM Tris-HCl pH 8.5, 500 mM NaCl). Resin was then washed extensively with column buffer and on-column cleavage was achieved by flushing the column with cleavage buffer (50 mM dithiothreitol, DTT in column buffer) and incubating overnight. rMucR, now cleaved from the poly-linker and carboxy-terminal intein-/chitin-binding domain, was recovered from the column by elution with column buffer. The concentration and purity of rMucR were assessed by absorbance measurements at 280 nm (A_280_) with a NanoDrop spectrophotometer and SDS-PAGE analysis.

### Electrophoretic mobility shift assays

EMSAs were carried out in 20 µL total reaction volumes using binding buffer comprised of 10 mM Tris-HCl (pH 7.4), 50 mM KCl, 1 mM DTT, 10% glycerol, and 0.05 mg/mL bovine serum albumin. PCR was used to generate DNA fragments corresponding to promoter regions using *B. abortus* 2308 gDNA and primers listed in Table S4. PCR fragments were then purified by gel electrophoresis and end-labeled with [λ- 32P] ATP (PerkinElmer, San Jose, CA, USA) and polynucleotide kinase (Promega, Madison, WI, USA). Increasing concentrations of rMucR and/or rMdrA were incubated with radiolabeled probes in binding buffer for 20 minutes at room temperature. rMucR purified from prMucR or pIB315 was used in EMSA reactions shown in Fig. S1 or Fig. 4, respectively. Homologous unlabeled DNA fragments were added to some reactions as specific competitors, and DNA fragments corresponding to the coding region of *emfA* (*bab_rs23470*) were added to others as non-specific competitors. Binding reactions were then electrophoresed on native 6% polyacrylamide gels in 0.5× TB (45 mM Tris base, 45 mM boric acid) running buffer for approximately 1 h at 100 volts. Gels were then visualized by autoradiography.

### ChIP-seq analysis

A recombinant version of the *Brucella* MucR purified from pIB315 was used to generate antiserum in rabbits (LabCorp, Madison, WI, USA), and this antiserum was used to detect MucR-binding sites in the *B. abortus* 2308 genome using the following procedures. Briefly, overnight cultures of *B. abortus* 2308 and an isogenic *mucR* mutant (CC092) were subcultured in 25 mL brucella broth and allowed to grow until early exponential phase (OD_600_ = 0.3). Cultures were then treated with 3% formaldehyde with rocking for 30 minutes at room temperature followed by quenching with glycine. Cells were centrifuged for 10 minutes at 14,000 × *g* in an Avanti J-E Series centrifuge (Beckman Coulter, Brea, CA, USA) at 4°C, washed twice with ice-cold PBS, and then resuspended in 0.5 mL ChIP Solution A (12.5 mM Tris pH 8, 12.5 mM EDTA pH 8, 62.5 mM NaCl, 25% sucrose) before being stored in liquid nitrogen. ChIP for *B. abortus* 2308 was performed similar to a previously described *Agrobacterium tumefaciens* protocol (71). Cells were lysed with 4 mg/mL lysozyme and sonicated using a Qsonica Q800R2 water bath sonicator. The chromosomal DNA was sheared to a size range of 70–330 bp, peaking at 150–160 bp (Fig. S4). The lysate was precleared using 50 µL beads (GE HealthCare Protein A Meg Sepharose, 28978111-AB), then incubated with 4 µL of anti-MucR antibodies at 4°C with rotation overnight. Then, lysates were incubated with the Protein A Meg Sepharose beads (28978111-AB) for 1 h at 4°C. After washes and elution, the immunoprecipitate was incubated at 65°C overnight to reverse the crosslinks. The DNA was further treated with RNaseA, proteinase K, extracted with phenol/chloroform/isoamylalcohol (25:24:1) (PCI), resuspended in 100 µL 0.1× Tris-EDTA (TE) buffer, and subjected to library preparation using the NEBNext Ultra II kit (E7645). The samples were sequenced using the Illumina NextSeq500 platform. The sequencing reads were aligned to the *B. abortus* 2308 reference genome (NCBI Reference Sequence GCA_000054005.1) using CLC Genomics Workbench (CLC Bio, QIAGEN). Sequencing reads from ChIP and input samples were normalized by the total number of reads for each sample. The ChIP enrichment (ChIP/Input) was plotted in R.

### Hi-C analysis

Overnight cultures of *B. abortus* 2308 and an isogenic *mucR* mutant (CC092) were subcultured in 25 mL brucella broth and allowed to grow until early exponential phase (OD_600_ = 0.3). Cultures were then treated with 3% formaldehyde with rocking for 30 minutes at room temperature followed by quenching with glycine. Cells were centrifuged for 10 minutes at 14,000 × *g* in an Avanti J-E Series centrifuge (Beckman Coulter, Brea, CA, USA) at 4°C, washed twice with ice-cold PBS, and then resuspended in 0.5 mL ice-cold PBS before being stored in liquid nitrogen. The detailed Hi-C procedure for *B. abortus* 2308 was adapted from a previously described *A. tumefaciens* protocol (71). Briefly, cells were lysed using Ready-Lyse Lysozyme (Epicentre, R1802M) followed by 0.5% SDS treatment. Solubilized chromatin was digested with DpnII for 2 h at 37°C. The digested ends were filled in with Klenow and Biotin-14-dATP, dGTP, dCTP, and dTTP. The products were ligated in dilute reactions with T4 DNA ligase at 16°C overnight. Crosslinks were reversed at 65°C overnight for about 20 h in the presence of EDTA, proteinase K, and 0.5% SDS. The DNA was then extracted twice with PCI, precipitated with ethanol, and resuspended in 20 µL of 0.1× TE buffer. Biotin from non-ligated ends was removed using T4 polymerase (4 h at 20°C) followed by extraction with PCI. The DNA was then sheared by sonication for 12 minutes with 20% amplitude using a Qsonica Q800R2 water bath sonicator. The sheared DNA was used for library preparation with the NEBNext Ultra II kit (E7645) following the manufacturer’s instructions for end repair, adapter ligation, and size selection. Biotinylated DNA fragments were purified using 5 µL streptavidin beads. DNA-bound beads were used for PCR in a 50 µL reaction for 14 cycles. PCR products were purified using Ampure beads (Beckman, A63881) and sequenced at the Indiana University Center for Genomics and Bioinformatics using NextSeq500. Paired-end sequencing reads were mapped to the combined genome files of *B. abortus* 2308 (NCBI Reference Sequence GCA_000054005.1) using the same pipeline described previously (71, 86). The combined *B. abortus* 2308 genome was divided into 656 5 kb bins. Subsequent analysis and visualization were done using R and Python scripts. To put *ori1* and *ori2* at the center, the reference genome of Ch1 starts at 950 kb and the genome of Ch2 starts at 550 kb.

### Complementation of *B. abortus* 2308 ∆*mucR* by *E. coli hns*

*B. abortus* 2308 and an isogenic *mucR* mutant (CC092) were transformed separately with either pSRKKm, pIB316, or pIB317 (Table S3), and the presence of the correct plasmids in the transformants was confirmed by PCR. Transformants were then grown to late exponential phase (OD_600_ = 1) in brucella broth, serially diluted, and then plated onto SBA in the presence or absence of 500 µM IPTG. Following 3-day incubation at 37°C, plates were examined for the formation of bacterial colonies.

RNA isolation and real-time (RT)-PCR analysis were used to assess the complementation of *btaE* overexpression in the *B. abortus mucR* mutant. Briefly, transformants were grown in brucella broth, then subcultured (OD_600_ = 0.1) in brucella broth in triplicate in the presence or absence of 1 mM IPTG. After growth to late exponential phase (OD_600_ = 1), total *Brucella* RNA was isolated as described previously (34). RNA was then treated with RNase-free DNase I (Ambion), and cDNA was generated using the SuperScript III cDNA synthesis system (Invitrogen, Carlsbad, CA, USA) following the manufacturer’s protocols. cDNA was then used for RT-PCR using a SYBR green PCR supermix (Roche, Mannheim, Germany). Primers for 16S RNA were used as a control along with *mucR*- and *btaE*-specific primers to evaluate relative mRNA levels (Table S4). PCR conditions were a single denaturing step (5 minutes at 95°C) followed by 40 cycles (denature for 15 seconds at 95°C, anneal for 15 seconds at 50°C, and extend for 15 seconds at 72°C). Fluorescence from SYBR green incorporation was measured with an iCycler machine (Bio-Rad), and the relative abundance of mRNA was determined using the 2^−ΔΔCt^ method.

## Data Availability

ChIP-seq and Hi-C data are deposited in the Gene Expression Omnibus (accession no. GSE234935).

## SUPPLEMENTAL MATERIAL

Figure S1 mbio.02201-23-s0001.tif EMSA analysis of rMucR binding.

Figure S2 mbio.02201-23-s0002.tif Normalized Hi-C contact frequency maps.

Figure S3 mbio.02201-23-s0003.tif Log_2_ ratio plot comparing *∆mucR* Hi-C matrices of two biological replicates.

Figure S4 mbio.02201-23-s0004.tif Distribution of fragment size from anti-MucR ChIP-seq results.

Supplemental material mbio.02201-23-s0005.docx Supplemental figure legends and Tables S1 to S4.

ASM does not own the copyrights to Supplemental Material that may be linked to, or accessed through, an article. The authors have granted ASM a non-exclusive, world-wide license to publish the Supplemental Material files. Please contact the corresponding author directly for reuse.
