# Supplementary figures and images for: Brucella MucR acts as an H-NS-like protein to silence virulence genes and structure the nucleoid
Source: mBio. 2023 Oct 17;14(6):e02201-23. doi: 10.1128/mbio.02201-23 (PMC10746212; doi:10.1128/mbio.02201-23)

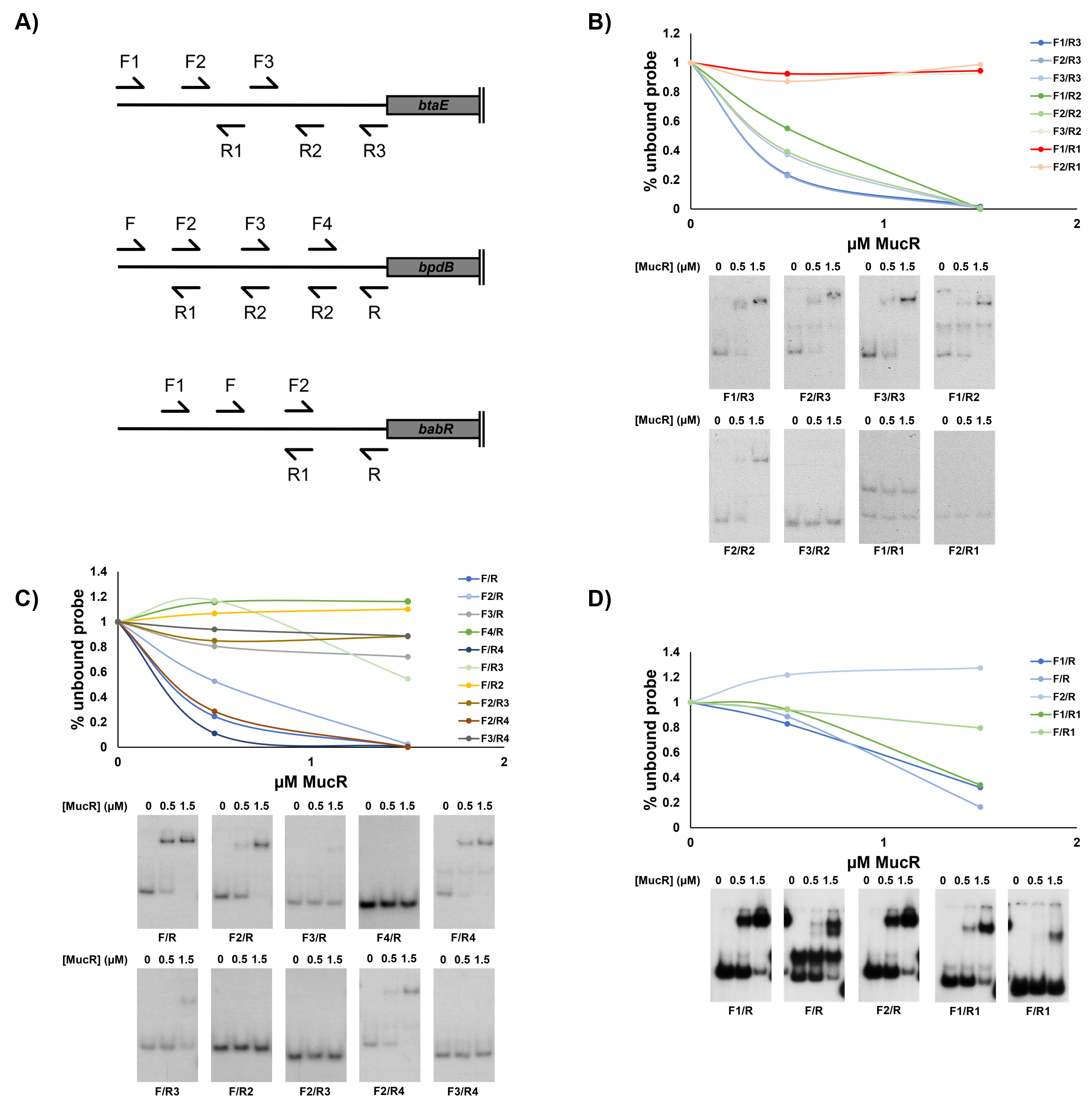

Supplement: Figure S1 — EMSA analysis of rMucR binding. [file mbio.02201-23-s0001.tif]

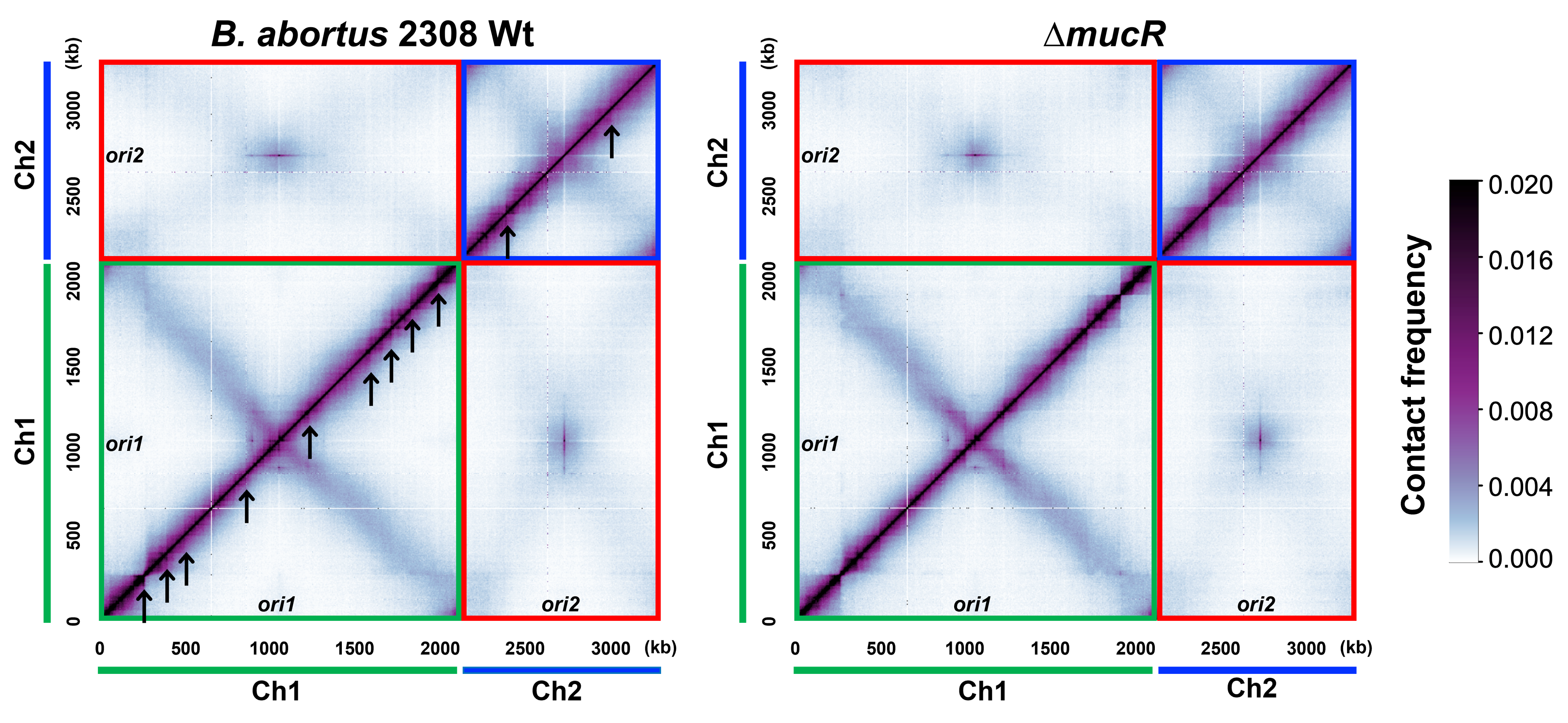

Supplement: Figure S2 — Normalized Hi-C contact frequency maps. [file mbio.02201-23-s0002.tif]

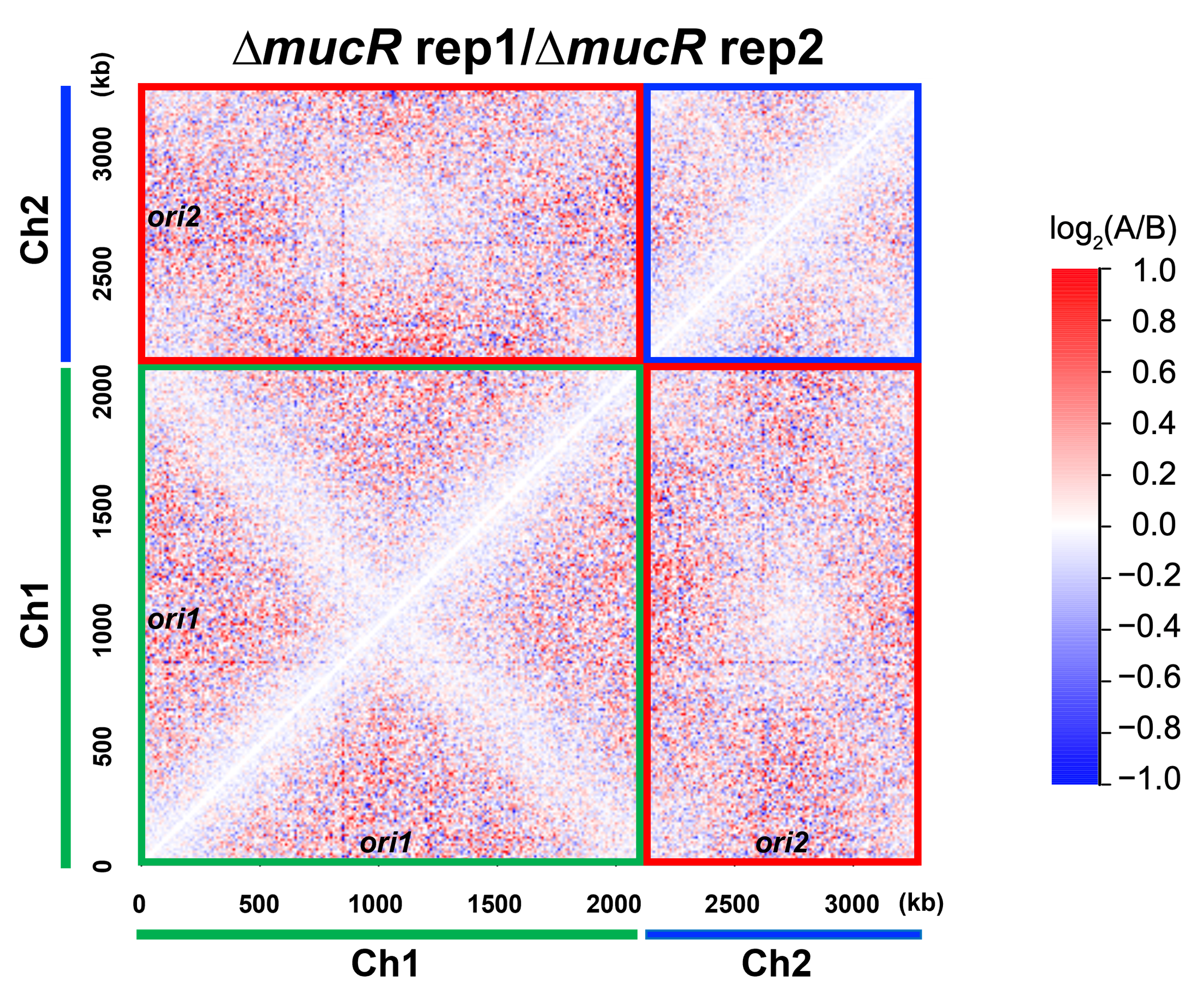

Supplement: Figure S3 — Log2 ratio plot comparing ∆mucR Hi-C matrices of two biological replicates. [file mbio.02201-23-s0003.tif]

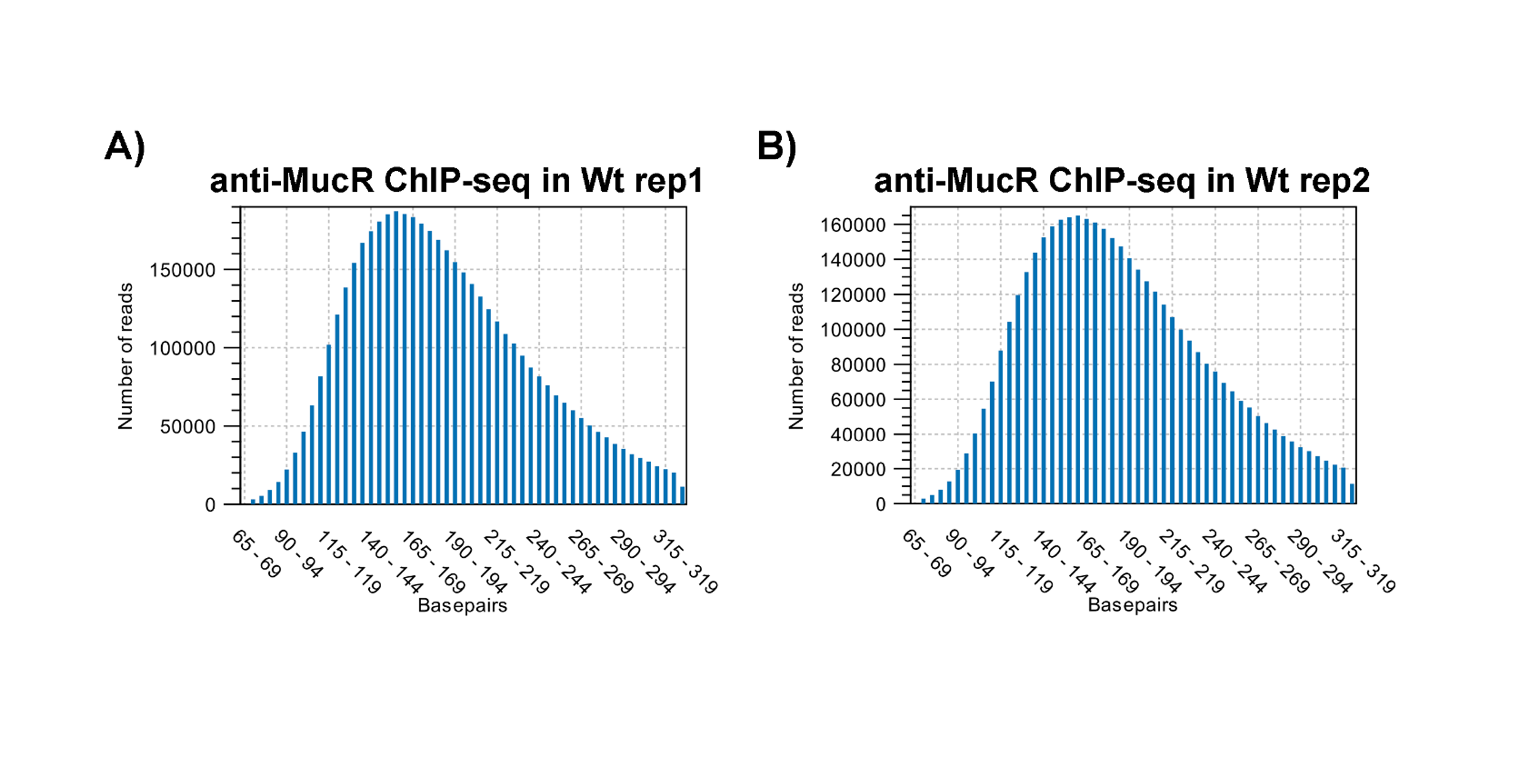

Supplement: Figure S4 — Distribution of fragment size from anti-MucR ChIP-seq results. [file mbio.02201-23-s0004.tif]
