# Supplementary material for: Brucella MucR acts as an H-NS-like protein to silence virulence genes and structure the nucleoid
Source: mBio. 2023 Oct 17;14(6):e02201-23. doi: 10.1128/mbio.02201-23 (PMC10746212; doi:10.1128/mbio.02201-23)
Supplement: Supplemental material — Supplemental figure legends and Tables S1 to S4. [file mbio.02201-23-s0005.docx]

**Figure S1.** EMSA analysis of rMucR binding to *btaE* (A,B), *bpdB* (A,C), and *babR* (A,D) promoter fragments. In B-C, densitometry analysis (above) of EMSA gels (below) shown as proportion of unbound probe relative to lane without rMucR. Relative positions of primers used to generate each fragment are shown in (A) and correspond to sequences listed in Table S4.

**Figure S2.** Normalized Hi-C contact frequency maps of the genomes of *B. abortus 2308* (left) and an isogenic *mucR* mutant (right). These are the same data as Figure 5A but plotted on a different color scale. The x-and-y axes correspond to genome position in kilobases (kb). The data are plotted in 5-kb bins. Chromosome 1 (Ch1, green bar), Chromosome 2 (Ch2, blue bar) and their respective origins (*ori1, ori2*) are labeled. Origins have been situated to the middle of each replicon for better visualization of the interactions within this region. The starting position for Ch1 is 950 kb and for Ch2 is 550 kb, but the Hi-C axes are shown as a contiguous number to represent the distance in kb. The scale bar for Hi-C interaction scores (contact frequency) is shown on the right. The Hi-C map can be divided into four parts: a Ch1 interaction map in the bottom left quadrant (green box), a Ch2 interaction map in the top right quadrant (blue box), and Ch1-Ch2 interaction map with identical, mirrored copies in the top left and bottom right quadrants (red boxes). On the Wt Hi-C map, black arrows point to a few examples of strong chromosomal interacting domain boundaries.

**Figure S3.** Log_2_ ratio plot comparing *∆mucR* Hi-C matrices of two biological replicates. Log_2_(matrix A/matrix B) was calculated and plotted in the heatmap. The color scale is shown.

**Figure S4.** Distribution of fragment size from anti-MucR ChIP-seq results. *B. abortus* 2308 Wt cells were subjected to anti-MucR ChIP-seq experiments in two biological replicates, rep1 in (A) and rep2 in (B). The ChIP-seq reads were mapped and analyzed using CLC genomics workbench. The distribution of fragment size were generated during the mapping process.

**Table S1: MucR binding sites within *B. abortus* 2308 as determined by ChIP-seq analysis**

| **Peak #** | **Chromosome** | **Peak Start**  **Position^a^** | **Peak End**  **Position^a^** | **Length (bp)** | **Peak shape score^a^** | **P-value^a^** |
| --- | --- | --- | --- | --- | --- | --- |
| 1 | 2 | 791335 | 792033 | 699 | 18.11 | 1.26E-73 |
| 2 | 1 | 582108 | 582647 | 540 | 17.76 | 6.65E-71 |
| 3 | 2 | 868960 | 869521 | 562 | 17.66 | 3.94E-70 |
| 4 | 1 | 272123 | 272786 | 664 | 17.63 | 7.00E-70 |
| 5 | 2 | 1086914 | 1087488 | 575 | 17.57 | 2.04E-69 |
| 6 | 1 | 1978466 | 1979076 | 611 | 17.40 | 4.04E-68 |
| 7 | 1 | 962870 | 963419 | 550 | 17.10 | 7.15E-66 |
| 8 | 2 | 133879 | 134419 | 541 | 17.07 | 1.23E-65 |
| 9 | 1 | 1545439 | 1545979 | 541 | 16.97 | 6.58E-65 |
| 10 | 1 | 15419 | 15969 | 551 | 16.82 | 9.35E-64 |
| 11 | 1 | 1089765 | 1090318 | 554 | 16.71 | 5.51E-63 |
| 12 | 1 | 964252 | 964979 | 728 | 16.67 | 1.11E-62 |
| 13 | 1 | 31966 | 32693 | 728 | 16.60 | 3.49E-62 |
| 14 | 1 | 1157370 | 1157909 | 540 | 16.55 | 7.67E-62 |
| 15 | 2 | 853447 | 853984 | 538 | 16.42 | 6.86E-61 |
| 16 | 2 | 942531 | 943092 | 562 | 16.41 | 8.09E-61 |
| 17 | 1 | 966223 | 966893 | 671 | 16.34 | 2.58E-60 |
| 18 | 1 | 1950517 | 1951074 | 558 | 16.33 | 3.04E-60 |
| 19 | 1 | 1462258 | 1462812 | 555 | 16.30 | 4.77E-60 |
| 20 | 2 | 674428 | 674965 | 538 | 16.23 | 1.58E-59 |
| 21 | 2 | 691618 | 692248 | 631 | 15.98 | 8.71E-58 |
| 22 | 1 | 736357 | 736916 | 560 | 15.97 | 1.09E-57 |
| 23 | 2 | 849178 | 849717 | 540 | 15.92 | 2.17E-57 |
| 24 | 1 | 255000 | 255634 | 635 | 15.86 | 6.19E-57 |
| 25 | 1 | 287672 | 288253 | 582 | 15.80 | 1.46E-56 |
| 26 | 2 | 491625 | 492225 | 601 | 15.79 | 1.72E-56 |
| 27 | 1 | 1067249 | 1067833 | 585 | 15.78 | 2.04E-56 |
| 28 | 1 | 729009 | 729553 | 545 | 15.75 | 3.63E-56 |
| 29 | 2 | 91792 | 92591 | 800 | 15.74 | 4.32E-56 |
| 30 | 2 | 677618 | 678416 | 799 | 15.71 | 6.25E-56 |
| 31 | 1 | 503188 | 503744 | 557 | 15.68 | 1.06E-55 |
| 32 | 1 | 543900 | 544421 | 522 | 15.59 | 4.48E-55 |
| 33 | 1 | 24070 | 24614 | 545 | 15.59 | 4.50E-55 |
| 34 | 1 | 1478922 | 1479478 | 557 | 15.54 | 9.06E-55 |
| 35 | 1 | 198746 | 199287 | 542 | 15.49 | 2.19E-54 |
| 36 | 1 | 2073574 | 2074125 | 552 | 15.48 | 2.40E-54 |
| 37 | 1 | 74617 | 75184 | 568 | 15.47 | 2.74E-54 |
| 38 | 1 | 1486448 | 1487011 | 564 | 15.44 | 4.39E-54 |
| 39 | 2 | 989110 | 989749 | 640 | 15.33 | 2.28E-53 |
| 40 | 2 | 1051242 | 1051814 | 573 | 15.24 | 1.02E-52 |
| 41 | 2 | 190582 | 191152 | 571 | 15.19 | 1.94E-52 |
| 42 | 1 | 644032 | 644721 | 690 | 15.13 | 5.31E-52 |
| 43 | 2 | 251758 | 252315 | 558 | 15.09 | 9.88E-52 |
| 44 | 1 | 1996050 | 1996590 | 541 | 14.94 | 9.34E-51 |
| 45 | 1 | 793804 | 794585 | 782 | 14.87 | 2.40E-50 |
| 46 | 2 | 766334 | 766882 | 549 | 14.82 | 5.54E-50 |
| 47 | 1 | 537257 | 537880 | 624 | 14.74 | 1.76E-49 |
| 48 | 1 | 1949780 | 1950399 | 620 | 14.74 | 1.90E-49 |
| 49 | 2 | 570661 | 571200 | 540 | 14.73 | 2.02E-49 |
| 50 | 2 | 601103 | 601641 | 539 | 14.68 | 4.14E-49 |
| 51 | 1 | 996742 | 997275 | 534 | 14.66 | 5.90E-49 |
| 52 | 1 | 1440291 | 1440915 | 625 | 14.65 | 7.13E-49 |
| 53 | 1 | 1551868 | 1552479 | 612 | 14.55 | 3.00E-48 |
| 54 | 1 | 1064424 | 1065016 | 593 | 14.52 | 4.80E-48 |
| 55 | 1 | 733822 | 734368 | 547 | 14.45 | 1.26E-47 |
| 56 | 1 | 1960993 | 1961540 | 548 | 14.41 | 2.29E-47 |
| 57 | 1 | 450822 | 451363 | 542 | 14.40 | 2.42E-47 |
| 58 | 2 | 860438 | 861237 | 800 | 14.37 | 3.77E-47 |
| 59 | 1 | 128637 | 129204 | 568 | 14.34 | 5.88E-47 |
| 60 | 1 | 323899 | 324482 | 584 | 14.27 | 1.76E-46 |
| 61 | 1 | 235128 | 235662 | 535 | 14.26 | 1.98E-46 |
| 62 | 2 | 604789 | 605330 | 542 | 14.26 | 2.00E-46 |
| 63 | 2 | 310521 | 311063 | 543 | 14.23 | 3.04E-46 |
| 64 | 2 | 128601 | 129400 | 800 | 14.21 | 4.13E-46 |
| 65 | 1 | 534380 | 535147 | 768 | 14.18 | 5.98E-46 |
| 66 | 1 | 1419973 | 1420533 | 561 | 14.14 | 1.08E-45 |
| 67 | 2 | 1100268 | 1100807 | 540 | 14.13 | 1.25E-45 |
| 68 | 2 | 1119416 | 1119953 | 538 | 14.03 | 5.02E-45 |
| 69 | 1 | 564385 | 564956 | 572 | 13.97 | 1.26E-44 |
| 70 | 1 | 1944646 | 1945444 | 799 | 13.93 | 2.07E-44 |
| 71 | 2 | 65744 | 66284 | 541 | 13.88 | 4.31E-44 |
| 72 | 1 | 93193 | 93726 | 534 | 13.86 | 5.89E-44 |
| 73 | 2 | 1103386 | 1103969 | 584 | 13.84 | 7.23E-44 |
| 74 | 1 | 547146 | 547728 | 583 | 13.83 | 8.69E-44 |
| 75 | 1 | 1959273 | 1959858 | 586 | 13.81 | 1.11E-43 |
| 76 | 2 | 447621 | 448157 | 537 | 13.74 | 2.97E-43 |
| 77 | 1 | 1702092 | 1702660 | 569 | 13.71 | 4.65E-43 |
| 78 | 1 | 1947242 | 1947909 | 668 | 13.68 | 6.52E-43 |
| 79 | 1 | 1938444 | 1939138 | 695 | 13.66 | 8.80E-43 |
| 80 | 1 | 2079928 | 2080469 | 542 | 13.59 | 2.40E-42 |
| 81 | 1 | 734703 | 735363 | 661 | 13.57 | 2.82E-42 |
| 82 | 1 | 1948488 | 1949194 | 707 | 13.56 | 3.69E-42 |
| 83 | 2 | 102884 | 103457 | 574 | 13.50 | 7.69E-42 |
| 84 | 2 | 865577 | 866125 | 549 | 13.46 | 1.27E-41 |
| 85 | 1 | 1730427 | 1730967 | 541 | 13.45 | 1.51E-41 |
| 86 | 1 | 325197 | 325742 | 546 | 13.45 | 1.54E-41 |
| 87 | 1 | 443628 | 444162 | 535 | 13.42 | 2.22E-41 |
| 88 | 1 | 1115677 | 1116232 | 556 | 13.40 | 2.85E-41 |
| 89 | 2 | 118811 | 119355 | 545 | 13.40 | 3.05E-41 |
| 90 | 1 | 535284 | 536068 | 785 | 13.39 | 3.37E-41 |
| 91 | 1 | 1792457 | 1793076 | 620 | 13.30 | 1.12E-40 |
| 92 | 1 | 1534081 | 1534651 | 571 | 13.24 | 2.72E-40 |
| 93 | 1 | 1254351 | 1254891 | 541 | 13.23 | 3.11E-40 |
| 94 | 1 | 1312636 | 1313205 | 570 | 13.17 | 6.35E-40 |
| 95 | 1 | 1943724 | 1944340 | 617 | 13.14 | 9.23E-40 |
| 96 | 2 | 553992 | 554542 | 551 | 13.10 | 1.66E-39 |
| 97 | 2 | 249127 | 249680 | 554 | 13.10 | 1.74E-39 |
| 98 | 2 | 54133 | 54766 | 634 | 13.05 | 3.34E-39 |
| 99 | 2 | 308819 | 309358 | 540 | 13.03 | 4.33E-39 |
| 100 | 2 | 836044 | 836652 | 609 | 13.02 | 4.84E-39 |
| 101 | 1 | 1331239 | 1331777 | 539 | 13.00 | 5.93E-39 |
| 102 | 2 | 833349 | 833893 | 545 | 12.99 | 7.07E-39 |
| 103 | 2 | 987926 | 988554 | 629 | 12.90 | 2.13E-38 |
| 104 | 1 | 957326 | 957874 | 549 | 12.81 | 7.05E-38 |
| 105 | 1 | 658598 | 659257 | 660 | 12.77 | 1.14E-37 |
| 106 | 1 | 46801 | 47384 | 584 | 12.69 | 3.17E-37 |
| 107 | 1 | 1951676 | 1952265 | 590 | 12.65 | 5.59E-37 |
| 108 | 1 | 1536817 | 1537378 | 562 | 12.65 | 5.83E-37 |
| 109 | 1 | 1953140 | 1953785 | 646 | 12.53 | 2.54E-36 |
| 110 | 1 | 206152 | 206705 | 554 | 12.52 | 3.05E-36 |
| 111 | 2 | 848339 | 848924 | 586 | 12.38 | 1.64E-35 |
| 112 | 1 | 1102296 | 1102900 | 605 | 12.35 | 2.47E-35 |
| 113 | 2 | 845464 | 846034 | 571 | 12.33 | 3.20E-35 |
| 114 | 1 | 545581 | 546159 | 579 | 12.18 | 1.96E-34 |
| 115 | 1 | 1060879 | 1061416 | 538 | 12.09 | 6.19E-34 |
| 116 | 1 | 552533 | 553097 | 565 | 11.99 | 2.00E-33 |
| 117 | 1 | 930083 | 930644 | 562 | 11.96 | 3.00E-33 |
| 118 | 1 | 538898 | 539676 | 779 | 11.94 | 3.63E-33 |
| 119 | 2 | 792383 | 792996 | 614 | 11.84 | 1.14E-32 |
| 120 | 2 | 466966 | 467511 | 546 | 11.78 | 2.54E-32 |
| 121 | 1 | 1802650 | 1803198 | 549 | 11.68 | 8.31E-32 |
| 122 | 2 | 735290 | 736088 | 799 | 11.63 | 1.37E-31 |
| 123 | 2 | 593410 | 593951 | 542 | 11.63 | 1.46E-31 |
| 124 | 1 | 1620156 | 1620715 | 560 | 11.61 | 1.93E-31 |
| 125 | 1 | 268669 | 269467 | 799 | 11.60 | 2.07E-31 |
| 126 | 1 | 336213 | 336757 | 545 | 11.54 | 3.94E-31 |
| 127 | 1 | 867497 | 868037 | 541 | 11.53 | 4.90E-31 |
| 128 | 1 | 723856 | 724393 | 538 | 11.48 | 8.33E-31 |
| 129 | 1 | 1427936 | 1428481 | 546 | 11.41 | 1.80E-30 |
| 130 | 2 | 571572 | 572268 | 697 | 11.38 | 2.65E-30 |
| 131 | 1 | 2081187 | 2081728 | 542 | 11.35 | 3.76E-30 |
| 132 | 2 | 812254 | 812807 | 554 | 11.32 | 5.23E-30 |
| 133 | 2 | 771656 | 772231 | 576 | 11.23 | 1.40E-29 |
| 134 | 1 | 1800476 | 1801071 | 596 | 11.01 | 1.68E-28 |
| 135 | 2 | 496238 | 496778 | 541 | 11.00 | 1.82E-28 |
| 136 | 2 | 503190 | 503733 | 544 | 10.99 | 2.06E-28 |
| 137 | 1 | 1727466 | 1728000 | 535 | 10.91 | 5.13E-28 |
| 138 | 2 | 575910 | 576490 | 581 | 10.86 | 8.70E-28 |
| 139 | 2 | 752628 | 753174 | 547 | 10.85 | 1.02E-27 |
| 140 | 1 | 207017 | 207573 | 557 | 10.80 | 1.80E-27 |
| 141 | 1 | 1808899 | 1809447 | 549 | 10.79 | 1.93E-27 |
| 142 | 2 | 1059274 | 1059811 | 538 | 10.61 | 1.41E-26 |
| 143 | 1 | 148819 | 149355 | 537 | 10.56 | 2.17E-26 |
| 144 | 1 | 1483603 | 1484173 | 571 | 10.53 | 3.28E-26 |
| 145 | 2 | 603904 | 604488 | 585 | 10.51 | 3.69E-26 |
| 146 | 1 | 663272 | 663857 | 586 | 10.43 | 9.17E-26 |
| 147 | 2 | 508340 | 508883 | 544 | 10.42 | 9.70E-26 |
| 148 | 2 | 707632 | 708391 | 760 | 10.39 | 1.33E-25 |
| 149 | 1 | 994605 | 995159 | 555 | 10.33 | 2.65E-25 |
| 150 | 2 | 374591 | 375201 | 611 | 10.30 | 3.47E-25 |
| 151 | 2 | 683755 | 684310 | 556 | 10.23 | 7.55E-25 |
| 152 | 1 | 1602658 | 1603243 | 586 | 10.21 | 9.08E-25 |
| 153 | 1 | 1568297 | 1568897 | 601 | 10.19 | 1.09E-24 |
| 154 | 2 | 610534 | 611111 | 578 | 10.17 | 1.35E-24 |
| 155 | 2 | 614902 | 615459 | 558 | 10.09 | 2.93E-24 |
| 156 | 1 | 1083970 | 1084535 | 566 | 10.06 | 4.18E-24 |
| 157 | 2 | 533712 | 534254 | 543 | 10.03 | 5.64E-24 |
| 158 | 1 | 1946189 | 1946744 | 556 | 10.02 | 6.49E-24 |
| 159 | 2 | 1122635 | 1123182 | 548 | 9.99 | 8.05E-24 |
| 160 | 1 | 928876 | 929449 | 574 | 9.97 | 1.07E-23 |
| 161 | 2 | 738871 | 739432 | 562 | 9.92 | 1.71E-23 |
| 162 | 1 | 1563166 | 1563724 | 559 | 9.87 | 2.91E-23 |
| 163 | 2 | 314705 | 315243 | 539 | 9.73 | 1.09E-22 |
| 164 | 1 | 1891233 | 1891778 | 546 | 9.70 | 1.55E-22 |
| 165 | 1 | 1729190 | 1729727 | 538 | 9.69 | 1.63E-22 |
| 166 | 1 | 275971 | 276509 | 539 | 9.68 | 1.75E-22 |
| 167 | 1 | 815654 | 816199 | 546 | 9.67 | 1.96E-22 |
| 168 | 2 | 916610 | 917159 | 550 | 9.59 | 4.44E-22 |
| 169 | 2 | 338259 | 338908 | 650 | 9.58 | 4.71E-22 |
| 170 | 1 | 548642 | 549179 | 538 | 9.58 | 4.78E-22 |
| 171 | 1 | 578492 | 579026 | 535 | 9.57 | 5.51E-22 |
| 172 | 1 | 1604883 | 1605418 | 536 | 9.49 | 1.13E-21 |
| 173 | 2 | 1023955 | 1024529 | 575 | 9.46 | 1.56E-21 |
| 174 | 2 | 1052262 | 1052806 | 545 | 9.44 | 1.95E-21 |
| 175 | 2 | 1057103 | 1057675 | 573 | 9.38 | 3.19E-21 |
| 176 | 1 | 1530734 | 1531273 | 540 | 9.34 | 4.91E-21 |
| 177 | 1 | 77316 | 78073 | 758 | 9.34 | 4.99E-21 |
| 178 | 1 | 881218 | 882016 | 799 | 9.34 | 5.03E-21 |
| 179 | 1 | 1806750 | 1807332 | 583 | 9.32 | 5.78E-21 |
| 180 | 2 | 767628 | 768237 | 610 | 9.21 | 1.69E-20 |
| 181 | 1 | 2066242 | 2066807 | 566 | 9.11 | 4.21E-20 |
| 182 | 1 | 201164 | 201697 | 534 | 9.05 | 7.15E-20 |
| 183 | 1 | 367476 | 368058 | 583 | 9.05 | 7.21E-20 |
| 184 | 2 | 990739 | 991399 | 661 | 9.04 | 7.53E-20 |
| 185 | 1 | 1692348 | 1692891 | 544 | 9.01 | 1.07E-19 |
| 186 | 2 | 385391 | 385938 | 548 | 8.96 | 1.56E-19 |
| 187 | 2 | 1143537 | 1144088 | 552 | 8.80 | 6.59E-19 |
| 188 | 2 | 428510 | 429046 | 537 | 8.80 | 7.02E-19 |
| 189 | 1 | 1059634 | 1060172 | 539 | 8.76 | 9.85E-19 |
| 190 | 1 | 1738911 | 1739506 | 596 | 8.73 | 1.28E-18 |
| 191 | 1 | 173989 | 174542 | 554 | 8.73 | 1.32E-18 |
| 192 | 1 | 1888178 | 1888728 | 551 | 8.72 | 1.44E-18 |
| 193 | 1 | 812677 | 813224 | 548 | 8.69 | 1.75E-18 |
| 194 | 1 | 327971 | 328520 | 550 | 8.63 | 3.14E-18 |
| 195 | 1 | 83404 | 83956 | 553 | 8.61 | 3.60E-18 |
| 196 | 1 | 1795919 | 1796466 | 548 | 8.60 | 3.98E-18 |
| 197 | 1 | 1616040 | 1616580 | 541 | 8.59 | 4.35E-18 |
| 198 | 1 | 1584174 | 1584836 | 663 | 8.57 | 5.37E-18 |
| 199 | 1 | 1621309 | 1621857 | 549 | 8.54 | 6.96E-18 |
| 200 | 2 | 311810 | 312391 | 582 | 8.49 | 1.03E-17 |
| 201 | 1 | 826726 | 827265 | 540 | 8.45 | 1.43E-17 |
| 202 | 1 | 727682 | 728367 | 686 | 8.39 | 2.41E-17 |
| 203 | 2 | 609362 | 609928 | 567 | 8.32 | 4.33E-17 |
| 204 | 2 | 672066 | 672608 | 543 | 8.30 | 5.25E-17 |
| 205 | 2 | 1145176 | 1145774 | 599 | 8.26 | 7.18E-17 |
| 206 | 1 | 570478 | 571050 | 573 | 8.22 | 1.03E-16 |
| 207 | 2 | 1043522 | 1044055 | 534 | 8.21 | 1.06E-16 |
| 208 | 1 | 1911819 | 1912379 | 561 | 8.19 | 1.31E-16 |
| 209 | 2 | 63578 | 64119 | 542 | 8.16 | 1.72E-16 |
| 210 | 1 | 1074871 | 1075425 | 555 | 8.10 | 2.82E-16 |
| 211 | 2 | 81480 | 82018 | 539 | 8.03 | 4.86E-16 |
| 212 | 2 | 643343 | 643889 | 547 | 8.02 | 5.23E-16 |
| 213 | 2 | 434196 | 434741 | 546 | 7.98 | 7.06E-16 |
| 214 | 2 | 346229 | 346824 | 596 | 7.98 | 7.35E-16 |
| 215 | 1 | 1799068 | 1799605 | 538 | 7.98 | 7.39E-16 |
| 216 | 1 | 60493 | 61043 | 551 | 7.91 | 1.28E-15 |
| 217 | 2 | 107691 | 108248 | 558 | 7.91 | 1.33E-15 |
| 218 | 2 | 382534 | 383115 | 582 | 7.90 | 1.44E-15 |
| 219 | 1 | 979211 | 979750 | 540 | 7.86 | 1.96E-15 |
| 220 | 1 | 457948 | 458520 | 573 | 7.81 | 2.96E-15 |
| 221 | 1 | 398986 | 399600 | 615 | 7.80 | 3.00E-15 |
| 222 | 1 | 1785940 | 1786510 | 571 | 7.77 | 3.95E-15 |
| 223 | 1 | 1624566 | 1625144 | 579 | 7.74 | 4.80E-15 |
| 224 | 1 | 1939746 | 1940288 | 543 | 7.74 | 4.86E-15 |
| 225 | 2 | 611974 | 612545 | 572 | 7.67 | 8.86E-15 |
| 226 | 2 | 247933 | 248484 | 552 | 7.66 | 9.39E-15 |
| 227 | 2 | 1142694 | 1143254 | 561 | 7.61 | 1.42E-14 |
| 228 | 1 | 1456949 | 1457671 | 723 | 7.55 | 2.21E-14 |
| 229 | 1 | 550116 | 550679 | 564 | 7.51 | 2.93E-14 |
| 230 | 1 | 1581708 | 1582273 | 566 | 7.42 | 5.93E-14 |
| 231 | 1 | 725500 | 726045 | 546 | 7.41 | 6.36E-14 |
| 232 | 1 | 1019608 | 1020141 | 534 | 7.39 | 7.31E-14 |
| 233 | 2 | 100098 | 100636 | 539 | 7.29 | 1.59E-13 |
| 234 | 2 | 1066969 | 1067503 | 535 | 7.27 | 1.84E-13 |
| 235 | 2 | 1044730 | 1045338 | 609 | 7.23 | 2.34E-13 |
| 236 | 2 | 592146 | 592695 | 550 | 7.19 | 3.26E-13 |
| 237 | 1 | 42260 | 42842 | 583 | 7.19 | 3.35E-13 |
| 238 | 2 | 1129359 | 1129895 | 537 | 7.18 | 3.52E-13 |
| 239 | 1 | 1586415 | 1587214 | 800 | 7.15 | 4.39E-13 |
| 240 | 1 | 1123595 | 1124166 | 572 | 7.11 | 5.64E-13 |
| 241 | 1 | 1942640 | 1943220 | 581 | 7.05 | 9.27E-13 |
| 242 | 2 | 117546 | 118238 | 693 | 7.04 | 9.73E-13 |
| 243 | 2 | 871563 | 872121 | 559 | 7.03 | 9.96E-13 |
| 244 | 1 | 1171524 | 1172145 | 622 | 7.03 | 9.99E-13 |
| 245 | 1 | 2070274 | 2070818 | 545 | 7.03 | 1.03E-12 |
| 246 | 2 | 1130372 | 1130906 | 535 | 7.01 | 1.16E-12 |
| 247 | 1 | 990847 | 991529 | 683 | 6.97 | 1.56E-12 |
| 248 | 1 | 1041862 | 1042436 | 575 | 6.93 | 2.12E-12 |
| 249 | 2 | 1067865 | 1068427 | 563 | 6.90 | 2.55E-12 |
| 250 | 1 | 1583269 | 1583810 | 542 | 6.90 | 2.57E-12 |
| 251 | 1 | 258056 | 258611 | 556 | 6.86 | 3.32E-12 |
| 252 | 1 | 401740 | 402351 | 612 | 6.85 | 3.57E-12 |
| 253 | 1 | 85273 | 86071 | 799 | 6.84 | 3.94E-12 |
| 254 | 1 | 1909801 | 1910340 | 540 | 6.84 | 3.97E-12 |
| 255 | 2 | 961231 | 961771 | 541 | 6.80 | 5.36E-12 |
| 256 | 1 | 689135 | 689703 | 569 | 6.77 | 6.33E-12 |
| 257 | 2 | 703407 | 703953 | 547 | 6.67 | 1.27E-11 |
| 258 | 1 | 2093281 | 2093826 | 546 | 6.63 | 1.73E-11 |
| 259 | 2 | 608162 | 608696 | 535 | 6.61 | 1.93E-11 |
| 260 | 2 | 151570 | 152144 | 575 | 6.60 | 2.10E-11 |
| 261 | 2 | 400266 | 400842 | 577 | 6.54 | 3.04E-11 |
| 262 | 2 | 162619 | 163161 | 543 | 6.53 | 3.28E-11 |
| 263 | 1 | 1147929 | 1148488 | 560 | 6.51 | 3.67E-11 |
| 264 | 1 | 1194959 | 1195525 | 567 | 6.51 | 3.70E-11 |
| 265 | 1 | 441849 | 442407 | 559 | 6.49 | 4.32E-11 |
| 266 | 1 | 471047 | 471586 | 540 | 6.48 | 4.51E-11 |
| 267 | 1 | 1735310 | 1735884 | 575 | 6.45 | 5.49E-11 |
| 268 | 1 | 329539 | 330096 | 558 | 6.42 | 6.77E-11 |
| 269 | 1 | 1438248 | 1438806 | 559 | 6.41 | 7.13E-11 |
| 270 | 2 | 1017830 | 1018363 | 534 | 6.35 | 1.10E-10 |
| 271 | 1 | 847528 | 848111 | 584 | 6.33 | 1.23E-10 |
| 272 | 2 | 880290 | 880853 | 564 | 6.30 | 1.51E-10 |
| 273 | 2 | 254506 | 255054 | 549 | 6.28 | 1.68E-10 |
| 274 | 2 | 74245 | 74797 | 553 | 6.26 | 1.89E-10 |
| 275 | 1 | 1404225 | 1404770 | 546 | 6.20 | 2.88E-10 |
| 276 | 2 | 619815 | 620352 | 538 | 6.15 | 3.88E-10 |
| 277 | 1 | 1308549 | 1309112 | 564 | 6.12 | 4.71E-10 |
| 278 | 1 | 62287 | 62920 | 634 | 6.11 | 4.99E-10 |
| 279 | 2 | 1141526 | 1142070 | 545 | 6.09 | 5.48E-10 |
| 280 | 1 | 1298660 | 1299216 | 557 | 6.08 | 6.17E-10 |
| 281 | 1 | 732176 | 732973 | 798 | 6.05 | 7.29E-10 |
| 282 | 1 | 473859 | 474415 | 557 | 6.05 | 7.36E-10 |
| 283 | 1 | 1256875 | 1257430 | 556 | 6.05 | 7.44E-10 |
| 284 | 2 | 528679 | 529224 | 546 | 6.03 | 8.42E-10 |
| 285 | 1 | 494821 | 495361 | 541 | 6.02 | 8.99E-10 |
| 286 | 2 | 224084 | 224626 | 543 | 6.00 | 9.83E-10 |
| 287 | 1 | 273861 | 274397 | 537 | 6.00 | 1.01E-09 |
| 288 | 2 | 908450 | 908984 | 535 | 5.99 | 1.08E-09 |
| 289 | 2 | 650594 | 651200 | 607 | 5.97 | 1.17E-09 |
| 290 | 1 | 632304 | 632850 | 547 | 5.97 | 1.19E-09 |
| 291 | 1 | 1769024 | 1769603 | 580 | 5.90 | 1.81E-09 |
| 292 | 2 | 554883 | 555514 | 632 | 5.89 | 1.91E-09 |
| 293 | 1 | 651942 | 652509 | 568 | 5.87 | 2.13E-09 |
| 294 | 1 | 624967 | 625522 | 556 | 5.87 | 2.15E-09 |
| 295 | 1 | 1721829 | 1722389 | 561 | 5.84 | 2.56E-09 |
| 296 | 1 | 1129655 | 1130210 | 556 | 5.84 | 2.62E-09 |
| 297 | 1 | 1000387 | 1000947 | 561 | 5.84 | 2.62E-09 |
| 298 | 2 | 1164 | 1728 | 565 | 5.82 | 2.90E-09 |
| 299 | 1 | 1940486 | 1941024 | 539 | 5.81 | 3.12E-09 |
| 300 | 1 | 1954823 | 1955452 | 630 | 5.79 | 3.53E-09 |
| 301 | 1 | 286240 | 286776 | 537 | 5.78 | 3.68E-09 |
| 302 | 2 | 305510 | 306056 | 547 | 5.74 | 4.66E-09 |
| 303 | 2 | 851626 | 852257 | 632 | 5.73 | 5.10E-09 |
| 304 | 1 | 541333 | 541888 | 556 | 5.72 | 5.35E-09 |
| 305 | 1 | 182838 | 183374 | 537 | 5.70 | 5.83E-09 |
| 306 | 2 | 896725 | 897264 | 540 | 5.68 | 6.56E-09 |
| 307 | 1 | 1003472 | 1004007 | 536 | 5.68 | 6.89E-09 |
| 308 | 2 | 996077 | 996706 | 630 | 5.59 | 1.10E-08 |
| 309 | 1 | 1573572 | 1574108 | 537 | 5.58 | 1.21E-08 |
| 310 | 1 | 311688 | 312222 | 535 | 5.57 | 1.30E-08 |
| 311 | 1 | 1607926 | 1608463 | 538 | 5.44 | 2.68E-08 |
| 312 | 2 | 8483 | 9029 | 547 | 5.43 | 2.81E-08 |
| 313 | 1 | 13496 | 14075 | 580 | 5.43 | 2.87E-08 |
| 314 | 1 | 649728 | 650342 | 615 | 5.41 | 3.15E-08 |
| 315 | 2 | 61603 | 62158 | 556 | 5.37 | 3.89E-08 |
| 316 | 2 | 858148 | 858682 | 535 | 5.37 | 4.01E-08 |
| 317 | 2 | 383448 | 383994 | 547 | 5.36 | 4.25E-08 |
| 318 | 1 | 530389 | 531188 | 800 | 5.35 | 4.34E-08 |
| 319 | 1 | 1673586 | 1674119 | 534 | 5.35 | 4.42E-08 |
| 320 | 1 | 1817289 | 1817915 | 627 | 5.32 | 5.11E-08 |
| 321 | 1 | 169083 | 169643 | 561 | 5.32 | 5.21E-08 |
| 322 | 1 | 626547 | 627206 | 660 | 5.31 | 5.34E-08 |
| 323 | 1 | 668412 | 668953 | 542 | 5.31 | 5.59E-08 |
| 324 | 1 | 783720 | 784324 | 605 | 5.30 | 5.86E-08 |
| 325 | 2 | 422632 | 423296 | 665 | 5.26 | 7.06E-08 |
| 326 | 1 | 1933155 | 1933909 | 755 | 5.20 | 9.74E-08 |
| 327 | 1 | 1372675 | 1373232 | 558 | 5.17 | 1.15E-07 |
| 328 | 1 | 2085132 | 2085698 | 567 | 5.17 | 1.16E-07 |
| 329 | 2 | 764851 | 765497 | 647 | 5.13 | 1.46E-07 |
| 330 | 1 | 1965340 | 1965896 | 557 | 5.10 | 1.72E-07 |
| 331 | 2 | 324038 | 324582 | 545 | 5.04 | 2.29E-07 |
| 332 | 1 | 278514 | 279071 | 558 | 5.01 | 2.75E-07 |
| 333 | 1 | 948007 | 948546 | 540 | 4.96 | 3.50E-07 |
| 334 | 2 | 239714 | 240513 | 800 | 4.93 | 4.01E-07 |
| 335 | 1 | 1599448 | 1600060 | 613 | 4.92 | 4.22E-07 |
| 336 | 1 | 1622565 | 1623119 | 555 | 4.92 | 4.34E-07 |
| 337 | 1 | 1146081 | 1146617 | 537 | 4.91 | 4.46E-07 |
| 338 | 1 | 1128815 | 1129363 | 549 | 4.90 | 4.67E-07 |
| 339 | 1 | 741208 | 741788 | 581 | 4.86 | 5.78E-07 |
| 340 | 1 | 2055406 | 2056010 | 605 | 4.84 | 6.45E-07 |
| 341 | 1 | 625876 | 626438 | 563 | 4.83 | 6.95E-07 |
| 342 | 1 | 1600410 | 1600999 | 590 | 4.80 | 7.78E-07 |
| 343 | 1 | 730408 | 730958 | 551 | 4.79 | 8.28E-07 |
| 344 | 1 | 106135 | 106673 | 539 | 4.77 | 9.13E-07 |
| 345 | 1 | 1223285 | 1223840 | 556 | 4.76 | 9.85E-07 |
| 346 | 1 | 170138 | 170728 | 591 | 4.75 | 9.95E-07 |
| 347 | 1 | 1099668 | 1100262 | 595 | 4.75 | 1.03E-06 |
| 348 | 1 | 1420822 | 1421480 | 659 | 4.74 | 1.07E-06 |
| 349 | 2 | 435388 | 435974 | 587 | 4.74 | 1.08E-06 |
| 350 | 1 | 1829280 | 1829847 | 568 | 4.72 | 1.17E-06 |
| 351 | 2 | 183458 | 184033 | 576 | 4.69 | 1.36E-06 |
| 352 | 1 | 1803538 | 1804143 | 606 | 4.68 | 1.47E-06 |
| 353 | 1 | 955193 | 955798 | 606 | 4.67 | 1.54E-06 |
| 354 | 2 | 984140 | 984744 | 605 | 4.66 | 1.55E-06 |
| 355 | 2 | 86940 | 87495 | 556 | 4.66 | 1.58E-06 |
| 356 | 1 | 448530 | 449067 | 538 | 4.64 | 1.77E-06 |
| 357 | 2 | 267442 | 268002 | 561 | 4.63 | 1.81E-06 |
| 358 | 2 | 1006355 | 1006972 | 618 | 4.62 | 1.92E-06 |
| 359 | 2 | 634340 | 634886 | 547 | 4.61 | 1.98E-06 |
| 360 | 2 | 1005263 | 1005796 | 534 | 4.57 | 2.43E-06 |
| 361 | 1 | 1001664 | 1002221 | 558 | 4.56 | 2.61E-06 |
| 362 | 1 | 330360 | 330895 | 536 | 4.54 | 2.77E-06 |
| 363 | 1 | 1439052 | 1439632 | 581 | 4.54 | 2.80E-06 |
| 364 | 1 | 954619 | 955193 | 575 | 4.51 | 3.29E-06 |
| 365 | 2 | 391954 | 392540 | 587 | 4.48 | 3.76E-06 |
| 366 | 2 | 321104 | 321646 | 543 | 4.47 | 3.84E-06 |
| 367 | 2 | 282003 | 282614 | 612 | 4.46 | 4.03E-06 |
| 368 | 1 | 574669 | 575266 | 598 | 4.46 | 4.16E-06 |
| 369 | 2 | 835039 | 835572 | 534 | 4.46 | 4.16E-06 |
| 370 | 2 | 178634 | 179241 | 608 | 4.44 | 4.55E-06 |
| 371 | 1 | 747509 | 748052 | 544 | 4.37 | 6.25E-06 |
| 372 | 1 | 1550542 | 1551189 | 648 | 4.36 | 6.59E-06 |
| 373 | 1 | 54752 | 55292 | 541 | 4.33 | 7.52E-06 |
| 374 | 1 | 1167061 | 1167622 | 562 | 4.31 | 8.22E-06 |
| 375 | 1 | 1529672 | 1530212 | 541 | 4.30 | 8.35E-06 |
| 376 | 2 | 96885 | 97426 | 542 | 4.30 | 8.59E-06 |
| 377 | 1 | 1625831 | 1626486 | 656 | 4.25 | 1.06E-05 |
| 378 | 1 | 758678 | 759238 | 561 | 4.24 | 1.10E-05 |
| 379 | 1 | 572069 | 572630 | 562 | 4.24 | 1.11E-05 |
| 380 | 1 | 2108178 | 2108717 | 540 | 4.22 | 1.23E-05 |
| 381 | 1 | 435544 | 436077 | 534 | 4.19 | 1.40E-05 |
| 382 | 2 | 663877 | 664411 | 535 | 4.16 | 1.57E-05 |
| 383 | 1 | 1017687 | 1018252 | 566 | 4.16 | 1.57E-05 |
| 384 | 2 | 157914 | 158454 | 541 | 4.16 | 1.59E-05 |
| 385 | 1 | 1971725 | 1972257 | 533 | 4.09 | 2.11E-05 |
| 386 | 2 | 648506 | 649039 | 534 | 4.09 | 2.12E-05 |
| 387 | 1 | 1797812 | 1798415 | 604 | 4.09 | 2.14E-05 |
| 388 | 2 | 101590 | 102167 | 578 | 4.09 | 2.15E-05 |
| 389 | 1 | 1896868 | 1897427 | 560 | 4.08 | 2.24E-05 |
| 390 | 1 | 583265 | 583885 | 621 | 4.08 | 2.26E-05 |
| 391 | 1 | 984965 | 985501 | 537 | 4.07 | 2.30E-05 |
| 392 | 2 | 232229 | 232813 | 585 | 4.07 | 2.31E-05 |
| 393 | 1 | 1771499 | 1772077 | 579 | 4.07 | 2.37E-05 |
| 394 | 2 | 1090885 | 1091446 | 562 | 4.01 | 2.98E-05 |
| 395 | 1 | 193435 | 194022 | 588 | 4.00 | 3.14E-05 |
| 396 | 1 | 2004960 | 2005589 | 630 | 3.97 | 3.60E-05 |
| 397 | 2 | 1053473 | 1054042 | 570 | 3.97 | 3.62E-05 |
| 398 | 1 | 767655 | 768233 | 579 | 3.97 | 3.66E-05 |
| 399 | 1 | 1988881 | 1989460 | 580 | 3.93 | 4.21E-05 |
| 400 | 1 | 512449 | 513100 | 652 | 3.92 | 4.49E-05 |
| 401 | 2 | 930326 | 930881 | 556 | 3.91 | 4.54E-05 |
| 402 | 2 | 618349 | 618920 | 572 | 3.88 | 5.28E-05 |
| 403 | 1 | 1813344 | 1813934 | 591 | 3.87 | 5.39E-05 |
| 404 | 2 | 682273 | 682837 | 565 | 3.86 | 5.61E-05 |
| 405 | 1 | 522363 | 522924 | 562 | 3.84 | 6.08E-05 |
| 406 | 2 | 714080 | 714775 | 696 | 3.82 | 6.78E-05 |
| 407 | 1 | 27382 | 27935 | 554 | 3.81 | 7.07E-05 |
| 408 | 1 | 1446639 | 1447172 | 534 | 3.79 | 7.51E-05 |
| 409 | 1 | 1856619 | 1857262 | 644 | 3.79 | 7.63E-05 |
| 410 | 1 | 1780383 | 1780952 | 570 | 3.78 | 7.73E-05 |
| 411 | 2 | 1146479 | 1147030 | 552 | 3.78 | 7.80E-05 |
| 412 | 1 | 1969170 | 1969736 | 567 | 3.78 | 7.84E-05 |
| 413 | 2 | 866589 | 867150 | 562 | 3.77 | 8.06E-05 |
| 414 | 1 | 913808 | 914345 | 538 | 3.76 | 8.66E-05 |
| 415 | 1 | 918685 | 919224 | 540 | 3.75 | 8.76E-05 |
| 416 | 1 | 790246 | 790854 | 609 | 3.74 | 9.17E-05 |
| 417 | 1 | 1634251 | 1634820 | 570 | 3.74 | 9.17E-05 |
| 418 | 1 | 969426 | 969977 | 552 | 3.72 | 9.90E-05 |
| 419 | 2 | 973685 | 974237 | 553 | 3.70 | 1.06E-04 |
| 420 | 1 | 920269 | 920820 | 552 | 3.68 | 1.15E-04 |
| 421 | 1 | 720447 | 721015 | 569 | 3.68 | 1.17E-04 |
| 422 | 1 | 1967152 | 1967685 | 534 | 3.66 | 1.28E-04 |
| 423 | 1 | 1500195 | 1500777 | 583 | 3.65 | 1.33E-04 |
| 424 | 1 | 859043 | 859691 | 649 | 3.63 | 1.42E-04 |
| 425 | 2 | 64693 | 65376 | 684 | 3.60 | 1.59E-04 |
| 426 | 1 | 742588 | 743162 | 575 | 3.60 | 1.61E-04 |
| 427 | 2 | 1098771 | 1099320 | 550 | 3.59 | 1.65E-04 |
| 428 | 2 | 662493 | 663127 | 635 | 3.59 | 1.65E-04 |
| 429 | 1 | 640691 | 641489 | 799 | 3.57 | 1.82E-04 |
| 430 | 2 | 361144 | 361693 | 550 | 3.56 | 1.87E-04 |
| 431 | 1 | 1913054 | 1913676 | 623 | 3.55 | 1.89E-04 |
| 432 | 1 | 1045790 | 1046346 | 557 | 3.55 | 1.91E-04 |
| 433 | 1 | 1796885 | 1797506 | 622 | 3.54 | 2.01E-04 |
| 434 | 1 | 1542056 | 1542855 | 800 | 3.53 | 2.07E-04 |
| 435 | 1 | 655182 | 655738 | 557 | 3.53 | 2.08E-04 |
| 436 | 2 | 675884 | 676481 | 598 | 3.52 | 2.16E-04 |
| 437 | 1 | 509252 | 509829 | 578 | 3.52 | 2.18E-04 |
| 438 | 1 | 475873 | 476425 | 553 | 3.51 | 2.20E-04 |
| 439 | 2 | 355840 | 356377 | 538 | 3.49 | 2.40E-04 |
| 440 | 1 | 1804538 | 1805337 | 800 | 3.48 | 2.46E-04 |
| 441 | 1 | 1026220 | 1026934 | 715 | 3.48 | 2.53E-04 |
| 442 | 1 | 25202 | 25740 | 539 | 3.47 | 2.58E-04 |
| 443 | 1 | 1454198 | 1454744 | 547 | 3.47 | 2.60E-04 |
| 444 | 1 | 307102 | 307684 | 583 | 3.47 | 2.63E-04 |
| 445 | 2 | 1078616 | 1079196 | 581 | 3.46 | 2.66E-04 |
| 446 | 1 | 1894976 | 1895510 | 535 | 3.42 | 3.10E-04 |
| 447 | 1 | 1371971 | 1372575 | 605 | 3.42 | 3.16E-04 |
| 448 | 1 | 989328 | 989918 | 591 | 3.42 | 3.17E-04 |
| 449 | 1 | 785979 | 786778 | 800 | 3.40 | 3.31E-04 |
| 450 | 1 | 1350081 | 1350646 | 566 | 3.40 | 3.34E-04 |
| 451 | 2 | 1041922 | 1042461 | 540 | 3.40 | 3.40E-04 |
| 452 | 2 | 830794 | 831346 | 553 | 3.39 | 3.52E-04 |
| 453 | 2 | 1101935 | 1102493 | 559 | 3.38 | 3.59E-04 |
| 454 | 1 | 246017 | 246561 | 545 | 3.29 | 4.97E-04 |
| 455 | 1 | 897245 | 897799 | 555 | 3.28 | 5.18E-04 |
| 456 | 1 | 1477894 | 1478470 | 577 | 3.26 | 5.48E-04 |
| 457 | 1 | 1150006 | 1150537 | 532 | 3.26 | 5.49E-04 |
| 458 | 1 | 1907124 | 1907923 | 800 | 3.24 | 5.93E-04 |
| 459 | 1 | 1970350 | 1970884 | 535 | 3.22 | 6.36E-04 |
| 460 | 1 | 440153 | 440785 | 633 | 3.20 | 6.86E-04 |
| 461 | 1 | 667441 | 667961 | 521 | 3.20 | 6.99E-04 |
| 462 | 1 | 1090849 | 1091631 | 783 | 3.19 | 7.14E-04 |
| 463 | 2 | 1002514 | 1003107 | 594 | 3.19 | 7.16E-04 |
| 464 | 1 | 225828 | 226373 | 546 | 3.19 | 7.23E-04 |
| 465 | 2 | 665484 | 666208 | 725 | 3.18 | 7.27E-04 |
| 466 | 2 | 1135891 | 1136445 | 555 | 3.18 | 7.32E-04 |
| 467 | 1 | 949333 | 949882 | 550 | 3.18 | 7.38E-04 |
| 468 | 2 | 964538 | 965148 | 611 | 3.17 | 7.66E-04 |
| 469 | 1 | 1513389 | 1514188 | 800 | 3.14 | 8.48E-04 |
| 470 | 2 | 393681 | 394242 | 562 | 3.14 | 8.58E-04 |
| 471 | 1 | 944375 | 944959 | 585 | 3.11 | 9.43E-04 |
| 472 | 2 | 1104645 | 1105252 | 608 | 3.10 | 9.80E-04 |
| 473 | 2 | 1007379 | 1007989 | 611 | 3.09 | 1.01E-03 |
| 474 | 1 | 1393932 | 1394461 | 530 | 3.07 | 1.08E-03 |
| 475 | 1 | 1956835 | 1957633 | 799 | 3.06 | 1.09E-03 |
| 476 | 2 | 66849 | 67487 | 639 | 3.06 | 1.09E-03 |
| 477 | 1 | 1935884 | 1936499 | 616 | 3.06 | 1.12E-03 |
| 478 | 2 | 1153671 | 1154231 | 561 | 3.06 | 1.13E-03 |
| 479 | 2 | 161547 | 162090 | 544 | 3.04 | 1.18E-03 |
| 480 | 2 | 692898 | 693509 | 612 | 3.03 | 1.23E-03 |
| 481 | 2 | 627811 | 628257 | 447 | 2.98 | 1.44E-03 |
| 482 | 1 | 1237979 | 1238566 | 588 | 2.98 | 1.44E-03 |
| 483 | 1 | 1564133 | 1564738 | 606 | 2.97 | 1.49E-03 |
| 484 | 1 | 373013 | 373612 | 600 | 2.97 | 1.51E-03 |
| 485 | 2 | 1156375 | 1156948 | 575 | 2.95 | 1.58E-03 |
| 486 | 2 | 398765 | 399424 | 660 | 2.93 | 1.71E-03 |
| 487 | 1 | 150373 | 150906 | 534 | 2.91 | 1.80E-03 |
| 488 | 1 | 527241 | 527851 | 611 | 2.91 | 1.83E-03 |
| 489 | 1 | 483474 | 484007 | 534 | 2.90 | 1.87E-03 |
| 490 | 1 | 1690647 | 1691183 | 537 | 2.90 | 1.88E-03 |
| 491 | 2 | 1034578 | 1035153 | 576 | 2.88 | 2.00E-03 |
| 492 | 2 | 1054855 | 1055423 | 569 | 2.88 | 2.01E-03 |
| 493 | 1 | 1997095 | 1997821 | 727 | 2.84 | 2.29E-03 |
| 494 | 2 | 951273 | 951843 | 571 | 2.83 | 2.30E-03 |
| 495 | 1 | 1307435 | 1307980 | 546 | 2.82 | 2.39E-03 |
| 496 | 2 | 1155092 | 1155636 | 545 | 2.80 | 2.58E-03 |
| 497 | 1 | 98953 | 99492 | 540 | 2.79 | 2.60E-03 |
| 498 | 1 | 124406 | 124952 | 547 | 2.79 | 2.64E-03 |
| 499 | 2 | 1149303 | 1149840 | 538 | 2.78 | 2.70E-03 |
| 500 | 1 | 992787 | 993387 | 601 | 2.78 | 2.70E-03 |
| 501 | 1 | 968598 | 969190 | 593 | 2.78 | 2.70E-03 |
| 502 | 1 | 1892944 | 1893490 | 547 | 2.78 | 2.71E-03 |
| 503 | 1 | 1012407 | 1012960 | 554 | 2.76 | 2.85E-03 |
| 504 | 1 | 2013380 | 2014082 | 703 | 2.72 | 3.27E-03 |
| 505 | 2 | 73256 | 73873 | 618 | 2.71 | 3.40E-03 |
| 506 | 2 | 855476 | 856155 | 680 | 2.69 | 3.57E-03 |
| 507 | 1 | 197051 | 197658 | 608 | 2.69 | 3.57E-03 |
| 508 | 1 | 1964460 | 1965011 | 552 | 2.68 | 3.71E-03 |
| 509 | 1 | 412731 | 413277 | 547 | 2.67 | 3.79E-03 |
| 510 | 1 | 1169878 | 1170397 | 520 | 2.66 | 3.94E-03 |
| 511 | 1 | 533049 | 533600 | 552 | 2.65 | 4.06E-03 |
| 512 | 1 | 1159657 | 1160232 | 576 | 2.63 | 4.28E-03 |
| 513 | 2 | 876228 | 876862 | 635 | 2.61 | 4.55E-03 |
| 514 | 2 | 1152760 | 1153301 | 542 | 2.61 | 4.56E-03 |
| 515 | 1 | 402727 | 403253 | 527 | 2.60 | 4.69E-03 |
| 516 | 1 | 1491221 | 1491867 | 647 | 2.59 | 4.76E-03 |
| 517 | 1 | 952549 | 953188 | 640 | 2.55 | 5.44E-03 |
| 518 | 2 | 88730 | 89315 | 586 | 2.53 | 5.65E-03 |
| 519 | 1 | 1048166 | 1048774 | 609 | 2.53 | 5.76E-03 |
| 520 | 1 | 2012723 | 2013363 | 641 | 2.52 | 5.83E-03 |
| 521 | 2 | 591250 | 591798 | 549 | 2.52 | 5.86E-03 |
| 522 | 1 | 1787068 | 1787604 | 537 | 2.50 | 6.23E-03 |
| 523 | 1 | 2044440 | 2044974 | 535 | 2.49 | 6.36E-03 |
| 524 | 1 | 738906 | 739480 | 575 | 2.49 | 6.42E-03 |
| 525 | 1 | 1975571 | 1976137 | 567 | 2.49 | 6.43E-03 |
| 526 | 2 | 1096615 | 1097236 | 622 | 2.49 | 6.44E-03 |
| 527 | 1 | 1934854 | 1935513 | 660 | 2.48 | 6.64E-03 |
| 528 | 2 | 688497 | 689148 | 652 | 2.47 | 6.68E-03 |
| 529 | 1 | 1031555 | 1032090 | 536 | 2.47 | 6.81E-03 |
| 530 | 1 | 1863634 | 1864167 | 534 | 2.44 | 7.29E-03 |
| 531 | 1 | 334460 | 335070 | 611 | 2.43 | 7.45E-03 |
| 532 | 1 | 1639655 | 1640141 | 487 | 2.43 | 7.46E-03 |
| 533 | 2 | 238375 | 238882 | 508 | 2.43 | 7.51E-03 |
| 534 | 1 | 1528676 | 1529295 | 620 | 2.43 | 7.61E-03 |
| 535 | 1 | 1991433 | 1991973 | 541 | 2.42 | 7.83E-03 |
| 536 | 1 | 961165 | 961706 | 542 | 2.41 | 8.07E-03 |
| 537 | 1 | 1484573 | 1485171 | 599 | 2.40 | 8.19E-03 |
| 538 | 1 | 82014 | 82601 | 588 | 2.39 | 8.44E-03 |
| 539 | 1 | 1225004 | 1225559 | 556 | 2.39 | 8.47E-03 |
| 540 | 1 | 1679237 | 1679776 | 540 | 2.38 | 8.59E-03 |
| 541 | 2 | 459409 | 459936 | 528 | 2.37 | 8.82E-03 |
| 542 | 1 | 949963 | 950553 | 591 | 2.37 | 8.84E-03 |
| 543 | 1 | 2111080 | 2111613 | 534 | 2.37 | 8.97E-03 |
| 544 | 1 | 28577 | 29120 | 544 | 2.35 | 9.26E-03 |
| 545 | 1 | 745586 | 746135 | 550 | 2.33 | 9.79E-03 |
| 546 | 1 | 500622 | 501300 | 679 | 2.33 | 9.85E-03 |

**^a^**ChIPseq start and end positions, peak shape score, and associated P-values as determined by CLC Genomics Workbench ^1^**.**

**Table S2: ChIP-seq overlap between MucR and VjbR^a^, CtrA^b^, and BvrR^c^ in *B. abortus* 2308**

| **Chr.** | **MucR peak #** | **Regulator**  **overlap(s)^d^** | **Associated**  **ORF(s)** | **Locus tag** | **UniProt Annotation** |
| --- | --- | --- | --- | --- | --- |
| 1 | 33 | V | BAB1_0021 |  | Pseudogene |
| 1 | 544 | B | BAB1_0025 | *rpsA* | 30S ribosomal protein S1 |
| 1 | 237 | V | BAB1_0037 | *moeA* | Molybdopterin molybdenumtransferase (EC 2.10.1.1) |
|  |  |  | BAB1_0038 | *cyoA* | Cytochrome oxidase subunit |
| 1 | 373 | C | BAB1_0047 | *ldt1* | Peptidoglycan L,D-transpeptidase |
| 1 | 72 | V, B | BAB1_0087 |  | DUF2794 domain-containing protein |
|  |  |  |  |  |  |
| 1 | 344 | C | BAB1_0102 | *phiA* | Peptidoglycan hydrolase inhibitor |
|  |  |  | BAB1_0103 |  | DUF218 domain-containing protein |
| 1 | 487 | C, B | BAB1_0137 |  | MmcB family DNA repair protein |
|  |  |  | BAB1_0138 | *ldt2* | Peptidoglycan hydrolase inhibitor |
| 1 | 346 | C | BAB1_0155 | *lptA* | LPS export |
| 1 | 464 | C | BAB1_0219 | *thiD* | Thiamine biosynthesis |
|  |  |  | BAB1_0220 |  | Bifunctional diguanylate cyclase/phosphodiesterase |
| 1 | 60 | V | BAB1_0324 |  | Hypothetical protein |
| 1 | 86 | V | BAB1_0325 |  | Solute-binding protein/glutamate receptor:Bacterial extracellular solute-binding protein, family 3 |
|  |  |  | BAB1_0326 |  | Glycosyl transferase, family 2 |
| 1 | 484 | B | BAB1_0373 |  | Extracytoplasmic solute receptor protein (TRAP transporter) |
| 1 | 221 | V | BAB1_0400 | *ygaE* | Aromatic amino acid transporter |
| 1 | 460 | C | BAB1_0444 | *pdxH* | Coenzyme metabolism |
|  |  |  | BAB1_0445 |  | Conserved 17kDa surface protein |
| 1 | 220 | V | BAB1_0467 |  | Peptidoglycan-binding LysM-type protein |
| 1 | 438 | C | BAB1_0484 | *acpP* | Acyl carrier protein (ACP) |
| 1 | 405 | V | BAB1_0528 |  | DUF1499 domain-containing protein |
|  |  |  | BAB1_0527 |  | Hypothetical protein |
| 1 | 511 | B | BAB1_0537 |  | No longer annotated |
| 1 | 69 | V | BAB1_0574 |  | Hypothetical protein |
| 1 | 379 | V | BAB1_0584 |  | Pseudogene |
|  |  |  | BAB1_0583 | *corA* | Mg^2+^ transporter |
| 1 | 290 | V | BAB1_0642 |  | DMT family transporter |
|  |  |  | BAB1_0641 | *pepN* | Aminopeptidase N (EC 3.4.11.2) |
| 1 | 42 | V | BAB1_0655 |  | Hypothetical protein |
| 1 | 314 | V, C | BAB1_0660 | *omp2b* | Porin |
| 1 | 435 | V, C | BAB1_0665 |  | No longer annotated |
| 1 | 524 | V, B | BAB1_0756 | *btpB* | Type 4 secretion effector protein |
| 1 | 324 | V | BAB1_0802 | *wcaJ* | Undecaprenyl-phosphate glucose phosphotransferase |
| 1 | 201 | V | BAB1_0852 | *ttcA* | tRNA modifcation |
| 1 | 127 | C | BAB1_0897 |  | Hypothetical protein |
| 1 | 333 | V | BAB1_0976 |  | No longer annotated |
| 1 | 542 | V, C | BAB1_0977 | *fumA* | Fumarate hydratase class I (EC 4.2.1.2) |
|  |  |  | BAB1_0978 | *ldt6* | Peptidoglycan L,D-transpeptidase |
| 1 | 418 | C | BAB1_1009 | *rlpA* | Endolytic peptidoglycan transglycosylase |
| 1 | 51 | V | BAB1_1035 |  | Hypothetical protein |
| 1 | 156 | C | BAB1_1119 | *coaD* | Coenzyme A biosynthesis |
| 1 | 347 | C | BAB1_1131 | *clpX* | ATP-dependent Clp protease ATP-binding subunit ClpX |
| 1 | 338 | C | BAB1_1159 | *ldt7* | Peptidoglycan L,D-transpeptidase |
| 1 | 457 | C | BAB1_1176 | *bamA* | Outer membrane protein assembly |
| 1 | 14 | C | BAB1_1185 |  | Pseudogene |
| 1 | 244 | V | BAB1_1201 |  | MerR-type transcriptional reguator |
|  |  |  | BAB1_1202 |  | Hypothetical protein |
| 1 | 482 | C | BAB1_1277 |  | PAS domain-containing protein |
| 1 | 94 | V | BAB1_1355 | *asp24* | Calcium-binding EF-hands protein |
| 1 | 66 | V | BAB1_1465 | *acm* | Peptidoglycan glycoside hydrolase |
|  |  |  | BAB1_1464 |  | Hypothetical protein |
| 1 | 52 | V | BAB1_1489 |  | Uncharacterized outer membrane protein |
|  |  |  | BAB1_1488 | *cgh* | Choloylglycine hydrolase (EC 3.5.1.24) |
| 1 | 537 | B | BAB1_1532 | *cspC* | Cold-shock DNA-binding domain |
| 1 | 38 | V | BAB1_1536 |  | Hypothetical protein |
|  |  |  | BAB1_1535 |  | Hypothetical protein |
| 1 | 423 | V | BAB1_1550 |  | DNA helicase |
|  |  |  | BAB1_1551 | *rplY* | 50S ribosomal protein L25 |
| 1 | 469 | B | BAB1_1563 |  | Purine nucleoside permease |
| 1 | 108 | V | BAB1_1587 | *lrp-2* | Leucine-responsive transcriptional regulator |
|  |  |  | BAB1_1586 | *trpE* | Anthranilate synthase (EC 4.1.3.27) |
| 1 | 153 | C | BAB1_1623 | *asmA* | Outer membrane protein assembly |
| 1 | 309 | B | BAB1_1627 | *potA* | Polyamine transport |
| 1 | 250 | V | BAB1_1639 | *omp31b* | OmpA-like protein |
| 1 | 267 | V | BAB1_1799 | *livH* | Branched chain amino acid transport |
| 1 | 291 | B | BAB1_1827 | *gdhZ* | NAD-dependent glutamate dehydrogenase |
| 1 | 352 | B | BAB1_2229 | *rrf* | 5S ribosomal RNA |
| 1 | 403 | C | BAB1_1867 | *ldt8* | Peptidoglycan L,D-transpeptidase |
| 1 | 350 | V, B | BAB1_1882 |  | MFS type transporter |
| 1 | 254 | B | BAB1_1966 | *metC* | Methionine biosynthesis |
| 1 | 208 | C | BAB1_1969 |  | No longer annotated |
| 1 | 527 | B | BAB1_1994 | *plsC* | Lipid transport and metabolism |
| 1 | 477 | B | BAB1_1995 | *ydcF* | Uncharacterized SAM-binding protein |
| 1 | 300 | B | BAB1_2016 | *rpmB* | 50S ribosomal protein L28 |
|  |  |  | BAB1_2017 |  | DUF3108 domain-containing protein |
| 1 | 422 | B | BAB1_2028 | *corB* | Mg^2+^ efflux |
| 1 | 385 | C | BAB1_2034 | *yafK* | Peptidoglycan L,D transpeptidase |
| 1 | 525 | V | BAB1_2037 | *dacC* | D-alanyl-D-alanine carboxypeptidase |
|  |  |  | BAB1_2038 |  | Metal dependent amidase |
| 1 | 36 | B | BAB1_2138 | *rlpA* | Endolytic peptidoglycan transglycosylase |
|  |  |  | BAB1_2139 |  | Hypothetical protein |
| 1 | 131 | C | BAB1_2147 | *cwlJ* | Cell wall hydrolase |
| 1 | 258 | C | BAB1_2159 | *hipB* | XRE-type transcriptional regulator |
| 2 | 312 | B | BAB2_0009 | *bmpA* | Adenine nucleotide translocator |
|  |  |  | BAB2_0010 |  | DUF922 domain-containing Zn-dependent protease |
| 2 | 71 | V | BAB2_0067 | *virB2* | Type IV secretion system protein VirB2 |
| 2 | 476 | V, B | BAB2_0068 | *virB1* | Type IV secretion system protein VirB1 |
| 2 | 211 | V | BAB2_0081 |  | RpoN-dependent transcriptional regulator |
|  |  |  | BAB2_0080 | *pepF* | Zinc metallopeptidase |
| 2 | 376 | B | BAB2_0097 |  | Amidase |
|  |  |  | BAB2_0098 |  | Ornithine/diaminopimelic acid/arginine decarboxylase |
| 2 | 233 | C | BAB2_0099 | *exoP* | Exopolysaccharide biosynthesis |
| 2 | 83 | C | BAB2_0104 |  | GT4 glycosyl transferase |
|  |  |  | BAB2_0105 |  | GT2 glycosyl transferase |
| 2 | 242 | B | BAB2_0118 | *vjbR* | Quorum sensing transcriptional regulator |
| 2 | 64 | C | BAB2_0131 | *lpxE* | Lipid A phosphatase |
|  |  |  | BAB2_0132 | *gtrA* | Cell surface polysaccharide synthesis |
| 2 | 334 | V | BAB2_0243 | *msrP* | Protein-methionine-sulfoxide reductase catalytic subunit |
|  |  |  | BAB2_0244 |  | Esterase |
| 2 | 273 | B | BAB2_0261 | *pntA* | Pyridine nucleotide transhydrogenase |
| 2 | 302 | V | BAB2_0310 | *puuR* | Polyamine-responsive transcriptional regulator |
|  |  |  | BAB2_0309 |  | Aspartate aminotransferase |
| 2 | 99 | V | BAB2_0313 | *lrp* | Leucine-responsive transcriptional regulator |
|  |  |  | BAB2_0312 | *alr* | Alanine racemase |
| 2 | 63 | V, C | BAB2_0314 |  | Uncharacterized OMP |
| 2 | 200 | B | BAB2_0315 | *lldD* | L-lactate dehydrogenase |
|  |  |  | BAB2_0316 | *mopB* | Molybdopterin oxidoreductase |
| 2 | 317 | V | BAB2_0388 |  | Hypothetical protein |
|  |  |  | BAB2_0389 |  | No longer annotated |
| 2 | 76 | C | BAB2_0450 |  | Hypothetical protein |
|  |  |  | BAB2_0451 | *dpdA* | Amino acid transport |
| 2 | 26 | V | BAB2_0500 |  | Pseudogene |
| 2 | 147 | V | BAB2_0516 |  | No longer annotated |
| 2 | 157 | C | BAB2_0535 | *sodC* | Superoxide dismutase [Cu-Zn] |
| 2 | 292 | V | BAB2_0558 |  | Amino acid transport |
| 2 | 130 | B | BAB2_0576 |  | DUF2938 domain-containing protein |
|  |  |  | BAB2_0577 |  | No longer annotated |
|  |  |  | BAB2_0578 |  | Xanthine/uracil/vitamin C permease |
| 2 | 145 | V | BAB2_0612 |  | Amino acid transport |
| 2 | 154 | V | BAB2_0622 |  | Pseudogene |
| 2 | 212 | V, C | BAB2_0652 | *lovK* | Blue-light-activated histidine kinase |
|  |  |  | BAB2_0653 |  | BA14K-like protein |
| 2 | 30 | V | BAB2_0681 |  | Pseudogene |
|  |  |  | BAB2_0680 |  | Hypothetical protein |
| 2 | 21 | C | BAB2_0695 |  | UDP-glucuronate decarboxylase |
|  |  |  | BAB2_0696 |  | No longer annotated |
| 2 | 148 | C | BAB2_0711 |  | Cytochrome oxidase assembly protein |
| 2 | 122 | V | BAB2_0738 |  | Monovalent cation/proton antiporter |
| 2 | 161 | V, C | BAB2_0741 | *mogA* | Molybdenum cofactor biosynthesis |
|  |  |  | BAB2_0742 |  | Hypothetical protein |
| 2 | 133 | V | BAB2_0782 | *def* | Peptide deformylase |
| 2 | 1 | V | BAB2_0806 |  | NarL/FixJ family transcriptional regulator |
|  |  |  | BAB2_0807 |  | Crp/Fnr family transcriptional regulator |
| 2 | 119 | V | BAB2_0808 |  | Hypothetical protein |
| 2 | 102 | V | BAB2_0847 |  | Hypothetical protein |
| 2 | 100 | C | BAB2_0850 | *cytC* | c-type cytochrome |
| 2 | 23 | V | BAB2_0862 | *hdeA* | Periplasmic acid stress chaperone |
| 2 | 15 | B | BAB2_0866 |  | Pseudogene |
|  |  |  | BAB2_0867 |  | Pseudogene |
| 2 | 184 | V, B | BAB2_1016 | *usp* | Universal stress protein |
| 2 | 40 | V | BAB2_1072 | *pemK* | MazF family toxin |
| 2 | 445 | V, C | BAB2_1099 | *ftcR* | Flagellar transcriptional regulator FtcR |
| 2 | 5 | C | BAB2_1106 | *fliC* | Flagellin |
|  |  |  | BAB2_1107 | *bmaC* | Autotransporter adhesin |
| 2 | 238 | B | BAB2_1136 |  | DUF1634 domain-containing protein |
|  |  |  | BAB2_1137 | *tauE* | Sulfite exporter |
| 2 | 411 | B | BAB2_1154 |  | Pseudogene |
| 2 | 478 | C | BAB2_1163 | *repA* | ParA plasmid-partitioning protein |
|  |  |  | BAB2_1161 | *hemN* | Heme biosynthesis |
| 2 | 485 | B | BAB2_1164 | *repB* | ParB plasmid-partitioning protein |

^a^VjbR ChIP-seq data taken from Kleinman *et al* 2017 ^28^.

^b^CtrA ChIP-seq data taken from Francis *et al* 2017 ^21^.

^c^BvrR ChIP-seq data taken from Rivas-Solano *et al* 2022 ^22^.

^d^Denotes ChIP peak position for VjbR (V), CtrA (C), and/or BvrR (B) falling within a given MucR ChIP peak (Table S1).

**Table S3: Plasmids used in this study**

| **Plasmid name** | **Description** | **Reference** |
| --- | --- | --- |
|  |  |  |
| pSRKKm | Broad host range cloning vector; IPTG-inducible; Km^R^ | Farrand *et al* 2008 ^84^ |
| prMucR | *B. abortus* 2308 *mucR* cloned into the expression vector pASK-IBA7+ with N-terminal Strep-tag; Ap^R^ | Caswell *et al* 2013 ^34^ |
| pIB314 | *B. abortus* 2308 *mdrA* cloned into the expression vector pASK-IBA7+ with N-terminal Strep-tag; Ap^R^ | This study |
| pIB315 | *B. abortus* 2308 *mucR* cloned into the expression vector pTXB1 with C-terminal polylinker fused to an intein/chitin binding domain | This study |
| pIB316 | *B. abortus* 2308 *mucR* cloned into pSRKKm | This study |
| pIB317 | *E. coli* MG1655 *hns* cloned into pSRKKm | This study |

**Table S4: Oligonucleotide primers used in this study**

| **Primer name** | **Sequence** | **Description** |
| --- | --- | --- |
| KP015 | gcgccgagaccgcggtcccgaattcatgaccaatacccagcgca | pASK-IBA7+ MdrA F |
| KP016 | cctgcaggtcgacctcgagggatccttacaggcggtaagcgatggag | pASK-IBA7+ MdrA R |
| IBP311 | tttaagaaggagatatacatatggaaaatctggaaac | pTXB1 MucR_F |
| IBP312 | tgcatctcccgtgatgcaggcgtccttcggcttgcgg | pTXB1 MucR_R |
| IBP325 | catatggaaaatctggaaacgaa | pSRK *mucR* F |
| IBP326 | aagcttaagaatcaggcgtccttcggcttgc | pSRK *mucR* R |
| IBP327 | catatgagcgaagcacttaaaat | pSRK *hns* F |
| IBP328 | aagcttaaagattattgcttgatcaggaaat | pSRK *hns* R |
| mucR qPCR F | ctgcttttgagtttgaccgc | *mucR* RT-qPCR primer |
| mucR qPCR R | gaacttcagcaatcagaaccg | *mucR* RT-qPCR primer |
| btaE qPCR F | caaggatgggattcggctac | *btaE* RT-qPCR primer |
| btaE qPCR R | gtctggtcggtataggctttc | *btaE* RT-qPCR primer |
| 16S qPCR F | tctcacgacacgagctgacg | 16s rRNA RT-qPCR primer |
| 16S qPCR R | cgcagaaccttaccagccct | 16s rRNA RT-qPCR primer |
| babR EMSA F1 | ccggttttatttttcgtccaaataaatattcg | *babR* EMSA probe |
| babR EMSA F2 | cttcgtagaagaaatatgaaagatatgc | *babR* EMSA probe |
| babR EMSA F3 | aaacggcaaaacagccctcatggagc | *babR* EMSA probe |
| babR EMSA F4 | tgtttgcttcaagctttgcggtgg | *babR* EMSA probe |
| babR EMSA F5 | ccggttttatttttcgtccaaataaatattcg | *babR* EMSA probe |
| babR EMSA R1 | gcatatctttcatatttcttctacgaag | *babR* EMSA probe |
| babR EMSA R2 | cactgtcggcagactgcatagcatca | *babR* EMSA probe |
| btaE EMSA F1 | gttcgattcctgttatttgt | *btaE* EMSA probe |
| btaE EMSA F2 | tataaataagaccgggaaaa | *btaE* EMSA probe |
| btaE EMSA F3 | gggatttacagattgggatt | *btaE* EMSA probe |
| btaE EMSA R1 | caatataaatttcttatatacatataatattg | *btaE* EMSA probe |
| btaE EMSA R2 | gaatatattttttaatttactccc | *btaE* EMSA probe |
| btaE EMSA R3 | gaccaggcatgtaaaatttt | *btaE* EMSA probe |
| bpdB EMSA F1 | gcgcatcttttattttagcct | *bpdB* EMSA probe |
| bpdB EMSA F2 | gcaaaattgtaaatattatagaagt | *bpdB* EMSA probe |
| bpdB EMSA F3 | tttaaccgtgaaataatgacaatta | *bpdB* EMSA probe |
| bpdB EMSA F4 | gtgatgccgaaaatatattcagact | *bpdB* EMSA probe |
| bpdB EMSA R1 | catctatatgtccacctaaaattcc | *bpdB* EMSA probe |
| bpdB EMSA R2 | acttctataatatttacaattttgc | *bpdB* EMSA probe |
| bpdB EMSA R3 | taattgtcattatttcacggttaaa | *bpdB* EMSA probe |
| bpdB EMSA R4 | agtctgaatatattttcggcatcac | *bpdB* EMSA probe |
